# Heterogeneously Assembled Metamaterials and Metadevices via 3D Modular Transfer Printing

**DOI:** 10.1038/srep27621

**Published:** 2016-06-10

**Authors:** Seungwoo Lee, Byungsoo Kang, Hohyun Keum, Numair Ahmed, John A. Rogers, Placid M. Ferreira, Seok Kim, Bumki Min

**Affiliations:** 1 SKKU Advanced Institute of Nanotechnology (SAINT) & School of Chemical Engineering, Sungkyunkwan University (SKKU), Suwon 16419, Republic of Korea; 2 Department of Mechanical Engineering, Korea Advanced Institute of Science and Technology (KAIST), Daejeon 34141, Republic of Korea; 3 Department of Mechanical Science and Engineering, University of Illinois, Urbana-Champaign, Illinois 61801, USA; 4 Department of Materials Science and Engineering, University of Illinois, Urbana-Champaign, Illinois 61801, USA

## Abstract

Metamaterials have made the exotic control of the flow of electromagnetic waves possible, which is difficult to achieve with natural materials. In recent years, the emergence of functional metadevices has shown immense potential for the practical realization of highly efficient photonic devices. However, complex and heterogeneous architectures that enable diverse functionalities of metamaterials and metadevices have been challenging to realize because of the limited manufacturing capabilities of conventional fabrication methods. Here, we show that three-dimensional (3D) modular transfer printing can be used to construct diverse metamaterials in complex 3D architectures on universal substrates, which is attractive for achieving on-demand photonic properties. Few repetitive processing steps and rapid constructions are additional advantages of 3D modular transfer printing. Thus, this method provides a fascinating route to generate flexible and stretchable 2D/3D metamaterials and metadevices with heterogeneous material components, complex device architectures, and diverse functionalities.

A range of exciting possibilities for molding the flow of electromagnetic waves has drawn immense interest in the field of “metamaterials”^1^,^2^,^3^,^4^,^5^,^6^,^7^,^8^,^9^,^10^,^11^,^12^,^13^. The theoretical and experimental investigations of negative refraction indices have been of particular interest, which have produced an explosion in the field of metamaterials over the last decade^1^,^2^,^3^,^4^,^5^,^6^,^9^,^11^. Moreover, various eccentric optical properties and functionalities, which are not natural, have been demonstrated, such as superlensing^3^, invisible cloaking (via transformation optics)^7^,^8^, gigantic chirality^14^,^15^, phase discontinuity control^16^,^17^, and zero and high refractive indices^10^,^12^,^13^. In the early stage, most attention was focused on the realization of these exotic electromagnetic properties both theoretically and experimentally. However, very recently, the field of metamaterials has been extended from the mere pursuit of such exotic electromagnetic properties towards the realization of practical devices^18^,^19^,^20^,^21^. For example, the concepts of dynamically-reconfigurable metadevices or functional metasurfaces have emerged and received much attention^18^,^19^,^20^,^21^,^22^,^23^. Therefore, the integration of heterogeneous constituting elements to form metadevices in diverse architectures is highly desirable^18^,^19^,^20^,^21^,^22^,^23^. For this purpose, each distinct meta-atom has to be put together in a single composite in either a two-dimensional (2D) or 3D configuration and integrated into a broad classes of substrates or active materials in a simple and robust manner.

Conventional methods for constructing metamaterials/metadevices heavily rely on monolithic micro/nanofabrication processes, such as photolithography, scanning beam lithography, physical/chemical deposition, and wet/dry etching^9^,^11^,^12^,^13^,^16^,^17^,^18^,^19^,^20^,^21^,^22^,^23^. These methods have been successful toolsets for the high-fidelity construction of 2D metamaterials/metadevices, but there are still significant drawbacks, especially for the 3D integration of heterogeneous materials onto universal substrates. (i) The high temperature and harsh chemical process may damage substrates or active materials where the metamaterials are fabricated. (ii) The complex and slow micro/nanofabrication process must be repeated until the meta-atoms and other functional materials are stacked in multiple 2D layers, which makes the 3D fabrication slow. (iii) The formation of each 2D layer without planarization, which makes the entire fabrication process even slower, places severe limitations on the quality and range of 3D device architectures and substrates. Only recently have authors reported that transfer printing may tackle the challenge of the fabrication of 3D metamaterials on a large area flexible substrate^24^. Despite being limited to bulk structures (i.e., a stacked fishnet), the latest successes of this work have demonstrated the feasibility of 2D or 3D assemblies of individual distinct meta-atom building blocks and dissimilar functional components on a wide range of unusual substrates.

A key goal of this paper is to demonstrate a method for integrating 2D/3D metamaterials/metadevices via *the 3D modular assembly of each distinct meta-atom building block* using an advanced mode of transfer printing. In contrast to traditional methodologies, 3D modular assembly via transfer printing enables the simple, robust, and versatile construction of various metamaterials by selecting desired subsets of meta-atoms and the subsequent 2D or 3D printing of them onto various substrates. Thus, the process throughput of our method (particularly for 3D construction) may be much higher than in traditional approaches. To demonstrate the efficiency of our method, we constructed four different types of architectures: (i) conventional 2D/3D metamaterial structures with designed photonic properties (e.g., fishnet, gammadion, I-beam, hexagonal frame, and cross), (ii) a stretchable metamaterial with an imbricate layout, (iii) a sensing metadevice attached to the surface of food, and (iv) electrically-gated graphene metadevices. Although this modular construction of heterogeneous 2D/3D metamaterials/metadevices is described for the operation at terahertz (THz) frequencies, the underlying principles of the method may be easily applicable to other frequency ranges (possibly up to mid-infrared frequencies) as long as the feature sizes of the modular components and the transfer printing processes are modified accordingly. For possible applications of the modular transfer printing technique at other frequency ranges, the minimum alignment error should be scaled accordingly, for which the resolution of vision optics and the precision of the translation stage would be the key enabling factors.

## Concept of 2D/3D modular transfer printing for metamaterials/metadevices

**Figure 1:**
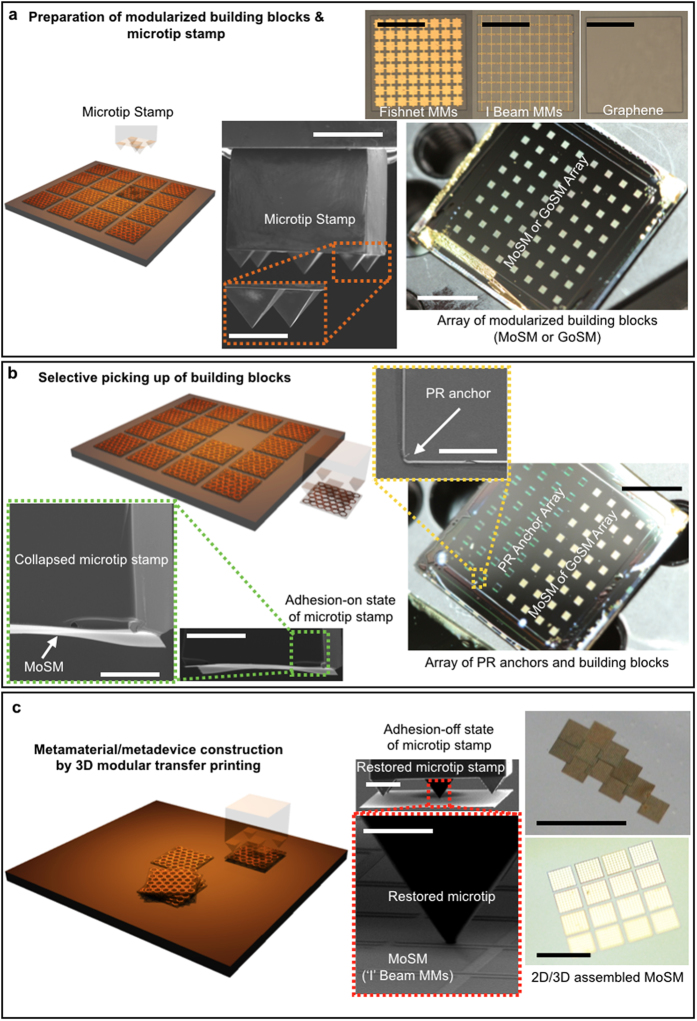
3D modular transfer printing for the assembly of heterogeneously integrated metamaterials/metadevices on universal substrates. (**a**) Preparation of the modularized basic building blocks (i.e., a metamaterial on a silicon (Si) membrane (MoSM) and graphene on a Si membrane (GoSM)) and an elastomeric 5-microtip stamp. The collective set of scanning electron microscope (SEM), optical microscope (OM), and macroscopic digital camera images shows MoSM, GoSM, the elastomeric 5-microtip stamp, and the MoSM/GoSM array on silicon (Si) wafers. The scale bars on the SEM images of the 5-microtip stamp are 300 μm and 100 μm (inset). All scale bars on the OM images (i.e., MoSM and GoSM) are 300 μm. The scale bar in the macroscopic digital camera image of the MoSM array is 6 mm. (**b**) Picking up of MoSM and GoSM using the 5-microtip stamp. The elastomeric 5-microtip stamp with a lateral dimension that is comparable to the size of MoSM and GoSM allows us to selectively pick up each individual building block. The macroscopic digital camera image and magnified SEM images show that each MoSM (bright square) can be selectively picked up by the 5-microtip stamp. The scale bars on the SEM images of the 5-microtip stamp are 200 μm and 100 μm (for the magnified image). The scale bars on the SEM image and macroscopic digital camera image of the PR anchors are 100 μm and 6 mm, respectively. (**c**) Construction of the metamaterial/metadevice via 3D modular transfer printing. Each individual building block (i.e., MoSM or GoSM) can be deterministically assembled into 2D and 3D metamaterials and metadevices. The scale bars on the SEM images of the 5-microtip stamp are 300 μm and 30 μm (magnified image). The scale bars on the OM images of the printed MoSM are 2.2 mm (top panel) and 1.4 mm (bottom panel), respectively.

The construction of the metamaterials/metadevices via 3D modular transfer printing was conducted in five representative steps: (i) define the on-demand metamaterial applications, (ii) design the metamaterials based on numerical simulations or theoretical considerations, (iii) fabricate the silicon (Si) membrane-based metamaterial building blocks (see Fig. 1a), (iv) select dissimilar substrates or functional materials with which the designed metamaterial building blocks are assembled, and (v) assemble each distinct metamaterial building blocks on the desired substrates or functional materials using 3D modular transfer printing (see Fig. 1b,c).

To test the method, a high resistivity float zone (HRFZ) silicon (Ultrasil Corp) membrane with a lateral dimension of 600 μm × 600 μm and a thickness of 2 or 3 μm was chosen as the basic building block. Optimal choice of a lateral dimension of the building block depends on factors such as overall footprints of metamaterials and metadevices, target applications, acceptable processing time, types of transfer printing machines, and sizes of each meta-atom. In the present work, for a proof of concept, we chose the lateral dimension of 600 μm by 600 μm for MoSM or GoSM in order to cover the minimum spot size of THz beam with 4 × 4 or 5 × 5 MoSM and GoSM arrays.

The doping level for silicon should be suppressed to maintain a relatively low absorption at the THz frequencies of interest (see Supplementary Fig. 1). To allow a specific optical function, the meta-atoms or graphene (as one of the representative active materials), which were pre-designed using numerical simulations, were incorporated onto the surface of a Si membrane (hereafter referred to as a metamaterial on a Si membrane, abbreviated as MoSM; graphene on a Si membrane, abbreviated as GoSM), as illustrated in Fig. 1a. These MoSM or GoSM building blocks were patterned and suspended on polymeric anchors using photolithography and controlled undercut etching of the silicon-on-insulator (SOI) wafers. After this initial preparation, the building blocks were ready for the subsequent selective retrieval steps (i.e., “inking” step in the transfer printing)^25^, as shown in Fig. 1b (details of the procedures are described in Supplementary Figs 2–4). All classes of the meta-atoms and other constituting elements used in this study are summarized in Supplementary Fig. 5.

For the heterogeneous assembly of MoSM and GoSM, the following three important requirements should be fulfilled. First, the construction should be based on non-specific surface interactions (i.e., van der Waals forces) to assemble dissimilar modular building blocks. Second, this assembly should be performed on a variety of substrates for a broad range of applications. Third, the modular building blocks should be assembled in a 3D fashion to enable more complex 3D metamaterial architectures.

To satisfy these three requirements, the modular assembly proposed in this work relies on transfer printing that uses an elastomeric 5-microtip stamp (made of polydimethylsiloxane (PDMS))^26^,^27^, which exhibits extremely high adhesion switchability (>1000) between the pressure-induced full collapsing mode with maximal contact (termed as “adhesion-on state”, presented in Fig. 1b) and the tip contact mode with minimal contact (“adhesion-off state”, presented in Fig. 1c). Detailed information on the elastomeric 5-microtip stamp regarding the material, dimension, and manufacturing is provided in the Supplementary Information (Supplementary Fig. 6). By collapsing the microtip stamp and successively retracting it with a high pulling speed, initially suspended MoSM or GoSM on polymeric anchors was successfully retrieved (with a yield approaching ~100% when properly prepared, see Fig. 1b). Immediately after the stamp was retracted, the collapsed microtip stamp returned to its original shape with a minimal contact area (i.e., the adhesion-off state) because of its restoring force (Fig. 1c). Conversely, this adhesion-off state along with the floppy property of the thin Si membrane (Fig. 1b) could facilitate a conformable dry contact between MoSM (or GoSM) and various materials of widespread surface roughness (successive retrieval and conformal printing are shown in Supplementary Fig. 7). Indeed, modular transfer printing of MoSM and GoSM was successfully performed on a wide range of materials, such as amorphous polymeric sheets, metallic films with different surface roughness, commercially available papers (with porous carbon fibril texture), and graphene and graphene oxide (see Supplementary Fig. 8). This transfer printing results clearly demonstrate unique assembling capabilities for the development of heterogeneously integrated 2D/3D metamaterials and metadevices.

### Assembly of 2D metamaterials onto universal substrates

**Figure 2:**
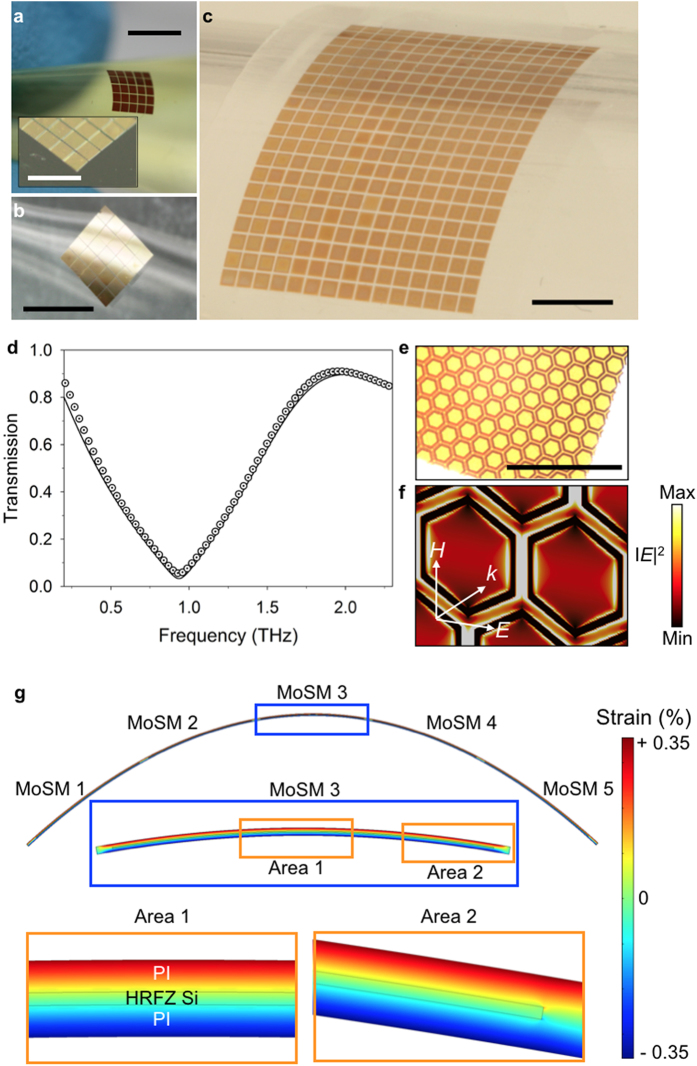
Development of 2D metamaterials on flexible and stretchable substrates via modular transfer printing. (**a**) A 5 × 5 printed honeycomb MoSM on a polyimide (PI) substrate. After modular transfer printing, the metamaterial was encapsulated by the PI. The scale bars are 3.5 mm and 2.0 mm (inset). (**b**) A 5 × 5 printed honeycomb MoSM on a stretchable polydimethylsiloxane (PDMS) substrate. The scale bar is 3.5 mm. (**c**) Large-scale (15 by 20) honeycomb MoSMs on PDMS assembled via the automated transfer printing machine. The scale bar is 3.5 mm. (**d**) Experimentally measured (circled dot) and simulated (solid line) terahertz (THz) amplitude transmittance of the assembled honeycomb metamaterial that was presented in (**a**). (**e**) Transmittance mode OM image of the honeycomb MoSMs. The scale bar is 300 μm. (**f**) Simulated spatial distribution of the electric field intensity (|*E*^2^|) at a resonance frequency (0.93 THz) of the honeycomb metamaterial. (**g**) Strain distribution in the bent Si membrane array (i.e., 5 × 5 array embedded within PI) with a radius curvature of 1.8 mm, which was calculated using the finite element method (FEM).

The aforementioned modular transfer printing technique was employed to assemble large-area 2D metamaterials. Because the transfer printing relies on van der Waals forces, the printed MoSM and GoSM can be retrieved and printed again as many times as possible before bonding irreversibly (see Supplementary Fig. 9). This reversible retrieval/printing enabled the adjustment of misaligned MoSM and GoSM, thereby minimizing the alignment error (below 5 μm) that was possibly caused by the vibration of the transfer printing machine. Therefore, modular printing allows MoSM and GoSM to be printed onto the substrate with a relatively regular spacing (see Supplementary Fig. 10). For more accurate THz spectroscopic measurements, a footprint of the metamaterials should be larger than the spot size of THz beam. Accordingly, we constructed 4 × 4 or 5 × 5 MoSM and GoSM arrays (distance between each building block was set to be between 30 μm and 50 μm). Figure 2a,b show representative examples of the 2D printed MoSM arrays (honeycomb metamaterials packed with hexagonal metallic frames) on a flexible amorphous polymer (polyimide (PI)) and a stretchable rubber (PDMS). A single cycle of the modular transfer printing, which consists of the retrieval, transferring, and printing of MoSM or GoSM onto the target position in the receiving substrate, was performed within approximately a minute even with a manual transfer printing machine (see Supplementary Fig. S11). Moreover, an automated machine (shown in Supplementary Fig. S12) provided large-area transfer printing of MoSM and GoSM arrays in a fast and precise manner (see Fig. 2c). Once the building blocks were prepared, a single layer metamaterial or metadevice with a THz measurable dimension was constructed in a substantially shorter time (less than ~30 min for manual printing and ~5 min for automated printing). For a more reliable operation, the printed MoSM and GoSM arrays need to be irreversibly bonded onto the receiving substrate because mechanical deformations (e.g., bending, twisting, or stretching) may result in the delamination of MoSM or GoSM from the receiving substrate. Therefore, as a final step, a permanent bonding procedure was performed by polymeric encapsulation and subsequent curing. The data from the THz spectroscopic measurements of the fabricated metamaterials were consistent with the simulated results (Fig. 2d); the transmission dip at 0.9 THz originated from the fundamental resonance of the honeycomb metamaterials^12^,^21^,^28^ (see the transmission optical microscope image of the structure in Fig. 2e and the simulated electric field intensity distribution at resonance in Fig. 2f). Figure 2g illustrates the finite-element simulation of strain in the bent PI/Si membrane composites (5 × 5 Si membrane array encapsulated in PI without meta-atom structures) with a 1.8 mm radius of curvature. All of the Si membranes embedded within the PI layers retained their structural fidelity under a high level of bending without any mechanical damage. The maximum strain in the composite was approximately ± 0.35%, which is below the fracture strain of Si (~1%). Thus, flexible metamaterials that are assembled via modular transfer printing can be robust with a certain degree of mechanical deformations.

The van der Waals force-enabled transfer printing of the modular components allows us to expand the range of possible applications of metamaterials because of the broad classes of usable substrates. For example, a functional MoSM array can be attached on food to implement a complete and passive-type sensor system (i.e., wireless food sensor)^29^. The variation in the surrounding dielectric condition affects the resonant behaviour of the honeycomb metamaterial via the change in the capacitive coupling between adjacent meta-atoms (hexagonal metallic frames of the honeycomb metamaterial). Thus, the metamaterials that are directly attached to food could function as RFID-like antennas for monitoring food quality (e.g., ripening status of cheese, as shown in Supplementary Fig. 13).

As another interesting example, MoSMs can also be integrated onto a stretchable PDMS post array with an imbricate geometry^30^ (i.e., partially overlapping MoSMs with metallic ‘I’ beam patterns, similar in appearance to fish or snake scales; see Supplementary Fig. 14a–e). Thus, full surface areal coverage by MoSMs can be ensured even under a stretched state (i.e., 17% stretching with respect to the initial state; see Supplementary Fig. 14f–h). This example of advanced stretchable metamaterials highlights the unique ability of the modular transfer printing strategy to expand the degrees of freedom in the design of metadevices, such as for wearable metamaterial antennas or sensors with flexibility and stretchability^28^.

### 3D Modular transfer printing of metamaterials

**Figure 3:**
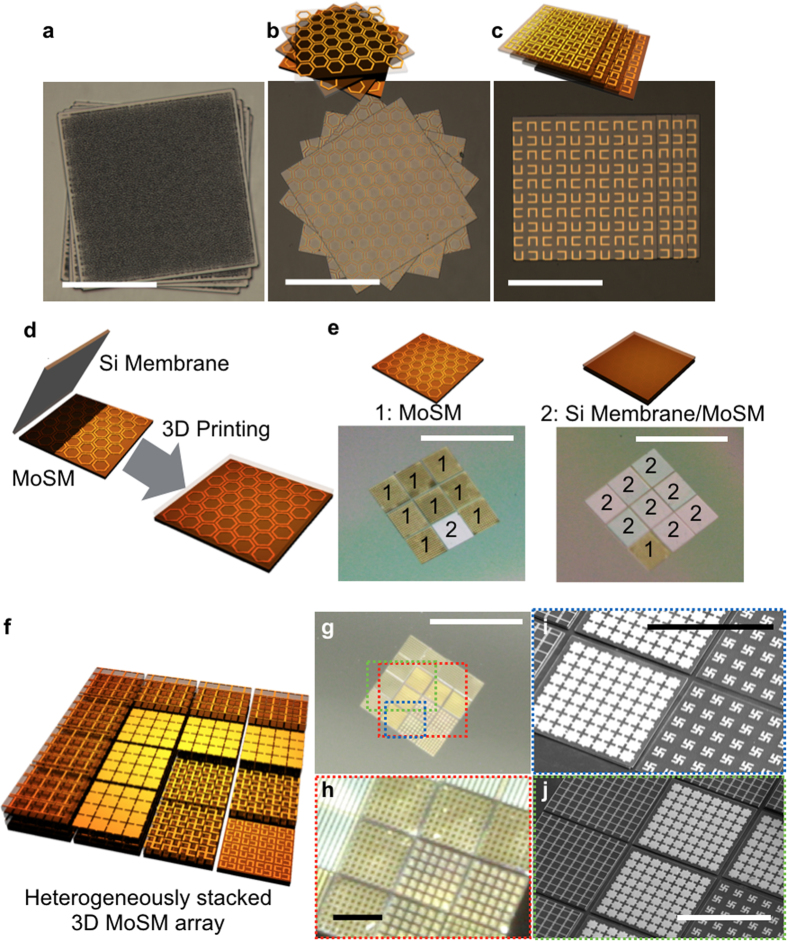
Deterministic construction via the 3D modular transfer printing of building blocks. (**a**) 3D printed rough silicon membranes (root mean square surface roughness is approximately 320 nm) each with a small rotation, (**b**) 3D printed honeycomb MoSMs each with a larger rotation, (**c**) 3D printed MoSM containing ‘U’ resonators twisted by 90° each with an incremental translation. All scale bars are 300 μm. (**d**) Schematic rendering of deterministic 3D printing of the Si membrane onto the previously printed MoSM, (**e**) Macroscopic digital camera images showing on-demand 3D modular printing of additional building blocks on any desired position. The MoSM is marked by ‘1’, and the Si membrane/MoSM stack is highlighted by ‘2’ in the macroscopic images. The Si membrane can be modularly printed onto any previously printed MoSM. All scale bars are 2 mm. (**f**) Schematic rendering of the 3D heterogeneous integration of different MoSMs. ‘U’ resonators twisted by 90°, gammadions, fishnet, and ‘I’ beam MoSMs are sequentially printed and stacked from the 1^st^ layer to the 4^th^ layer. (**g**) Macroscopic digital camera image of the heterogeneously stacked 5 × 5 MoSM array that is schematically illustrated in (**f**). The scale bar is 2.5 mm. (**h**) Magnified image of the red dotted box in (**g**). The scale bar is 500 μm. (**i**) Magnified SEM image of the blue dotted box in (**g**), (**j**) Magnified SEM image of green dotted box in (**g**). All scale bars are 600 μm.

Various MoSM building blocks can be assembled into 3D architectures with a relatively high precision and flexibility (in terms of spatial arrangement of building blocks, number of stacked layers, and heterogeneous combination of different MoSMs), which is a prerequisite for attaining desired electromagnetic behaviours from 3D metamaterials. Figure 3 highlights the capability of assembling the building blocks in 3D layouts with a high precision. To be more specific, the relative alignment errors between MoSMs were kept below 5 μm (see Supplementary Fig. 15) during 3D transfer printing (i.e., 3D stacking of MoSMs). Excessive accumulation of alignment errors during the stacking could be prevented because newly transferred MoSMs were always aligned with respect to the first transferred MoSMs. The demonstrated 3D architectures in this work include (i) 4 stacked rough Si membranes (root mean square surface roughness was approximately 320 nm) with incremental rotations (Fig. 3a), (ii) 4 stacked MoSMs (honeycomb metamaterials) with incremental rotations (Fig. 3b), (iii) 4 stacked MoSMs (‘U’ resonators twisted by 90°) with incremental translations (Fig. 3c), (iv) precisely controlled 3D modular printing of MoSMs onto a previously printed 3 × 3 MoSM array (Fig. 3d,e), and (v) 3D stack of heterogeneous MoSMs (from 1^st^ to 4^th^ layer: ‘U’ resonators twisted by 90°, Gammadions, fishnet, and ‘I’ beams, as shown in Fig. 3f–j). The construction of this 3D metamaterial was rapidly performed (on the order of a minute per each MoSM); this unique advantage of high processing speed can be more pronounced, as the number of the stacked layer is increased. To ensure a strong adhesion between the vertically stacked MoSMs, we used thermal annealing (330 °C for 2 hour) and encapsulated the stacked MoSMs with PI (see Supplementary Fig. 16). This 3D modular printing makes it highly appealing for the assembly of 3D metamaterials that consist of heterogeneous meta-atoms subsets.

**Figure 4:**
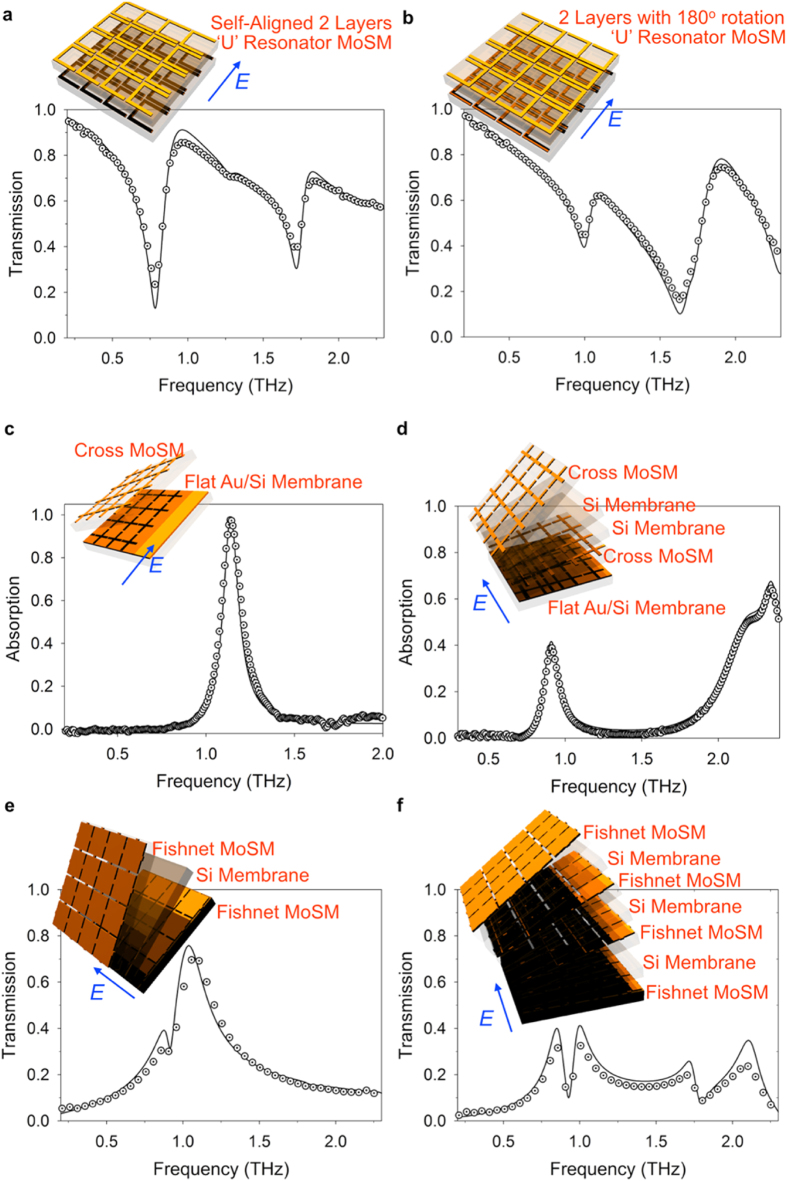
THz response of the assembled 3D metamaterials constructed via modular transfer printing. (**a**) Stacked ‘U’ resonators in a self-aligned manner (total 2 layers), (**b**) Stacked ‘U’ resonators with a 180° rotation angle (total 2 layers). (**c**) Metamaterial perfect absorber with stacked cross MoSMs onto a flat Au membrane (i.e., flat Au on Si membrane) (total 2 layers), (**d**) Metamaterial absorber with a multi-layered cross MoSM/Si membrane/Si membrane/cross MoSM/flat Au membrane (total 5 layers). (**e**) Stacked fishnet metamaterials: fishnet MoSM/Si membrane/fishnet MoSM, (**f**) Multi-layered fishnet (total 7 layers): fishnet MoSM/Si membrane/fishnet MoSM/Si membrane/fishnet MoSM/Si membrane/fishnet MoSM. All of these metamaterials were developed by 3D modular transfer printing and were embedded within a 15 μm thick PI layer. The electric field direction of the incident wave is indicated by the blue arrows, and the propagation direction of the incident wave is normal to the surface of the constructed metamaterials.

The THz amplitude transmittance through 3D metamaterials that were assembled via 3D modular transfer printing is shown in Fig. 4a–f. To ensure the generality, various 3D metamaterials were tested, such as (i) stereo metamaterials (realized by stacking ‘U’ resonators with controlled rotational angles between the layers; 2 layers of MoSMs in total, as shown in Fig. 4a,b), (ii) metamaterial absorbers (realized by stacking MoSM with metallic cross patterns and a flat Au/Si membrane; 2 layers of MoSMs in total, as shown in Fig. 4c. In addition, Fig. 4d shows the data from a multi-layered structure consisting of a MoSM with metallic cross patterns, two Si membrane, another MoSM with metallic cross patterns, and a flat Au/Si membrane; 5 layers of MoSMs in total), and (iii) a stacked fishnet (i.e., fishnet MoSM/Si membrane/fishnet MoSM; 3 layers of MoSMs in total, as shown in Fig. 4e. In addition, Fig. 4f shows a multi-layered stack of a fishnet MoSM/Si membrane; 7 layers of MoSMs in total). As with the 2D assembly, these 3D metamaterials can also be assembled onto various substrates. For example, metamaterial absorbers (Supplementary Fig. 17a) were assembled onto a flat metallic substrate (see Supplementary Fig. 17b–d) or on a THz-transparent PDMS rubber substrate (see Supplementary Fig. 17e). Furthermore, even with a 5-μm scale misalignment of MoSMs, which occurred particularly in our manual printing process (Supplementary Fig. 11), the response of the THz metamaterials was quite robust against this alignment error. Indeed, the experimentally measured THz transmission spectra show excellent agreement with the theoretical prediction, as observed in Fig. 4. The precise control of certain properties of the metamaterials was achieved easily by adjusting several controllable factors, such as the type of MoSMs, the number of assembled layers, the spacing distance between layers, and the translational shifts and rotational angles between the MoSM layers. Therefore, we proposed a possible methodology for assembling 3D heterogeneous metamaterials using the fast and simple 3D modular printing technique, which may be useful for on-demand engineering of sophisticated electromagnetic properties of metamaterials.

### Assembly of graphene metadevices via 3D modular transfer printing

**Figure 5:**
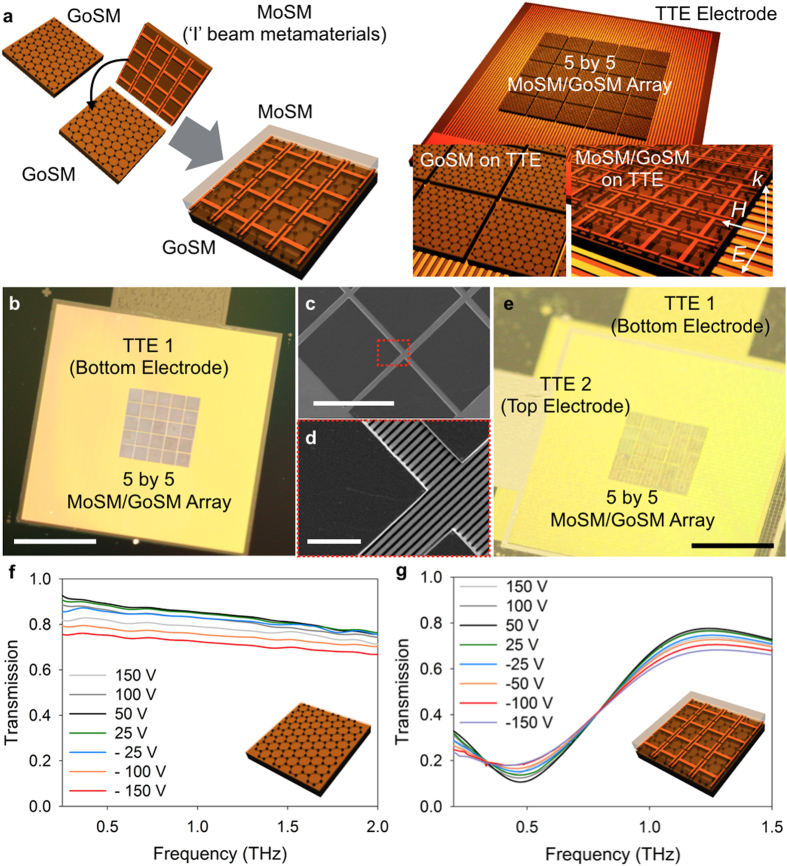
Assembly of active metadevices via 3D modular transfer printing of MoSM and GoSM. (**a**) Schematic rendering of the assembly of different building blocks (MoSM, GoSM, and THz transparent electrode (TTE)) via 3D modular transfer printing. (**b**) Macroscopic digital camera image of the printed 5 × 5 MoSM/GoSM array on the TTE. The scale bar is 3.5 mm. (**c**) Magnified SEM image of (**b**). The scale bar is 600 μm. (**d**) High-magnification image of (**c**). The scale bar is 80 μm. (**e**) Macroscopic digital camera image of the assembled graphene metadevice (from the bottom to the top: TTE, GoSM, MoSM, adhesive layer, and TTE). The scale bar is 3.5 mm. (**f**) Experimentally measured THz amplitude transmittance curves for the graphene device (without metamaterials) as a function of gate-voltage. (**g**) Experimentally measured THz amplitude transmittance curves for the graphene metadevice as a function of gate-voltage.

To further elucidate the capability of 3D heterogeneous modular assembly for the construction of metamaterials and metadevices, we assembled electrically-gated graphene metadevices that consist of three main building blocks: (i) a MoSM with metallic ‘I’ beam patterns, (ii) a THz transparent electrode (TTE) layer, and (iii) a GoSM as an active component for electrically-controlled metadevices. Electrically-gated graphene metadevices provide an electrical means for the control of the amplitude and phase of the transmitted THz wave^21^. Figure 5a shows a schematic of the fabrication process for electrically-gated graphene metadevices via 3D modular transfer printing. The printing of each modularized component can reduce the process complexity of the 3D heterogeneous integration. To show the versatility of the process for the construction of electrically-gated graphene metadevices, we constructed two different assemblies including (i) GoSM/TTE and (ii) MoSM/GoSM/TTE for a simple graphene-only modulator and a graphene metamaterial modulator.

To gate the graphene uniformly over the large area, we introduced a specific type of gate-electrode (i.e., TTE) that we had previously reported^21^. In particular, TTE is an array of Au microwire with a metallic width of 5 μm and a periodicity of 10 μm, and it is designed to be transparent for linearly-polarized THz waves (the polarization of which is parallel to the grating vector of the microwire array) for the frequency of interest (see Supplementary Fig. 18). It simultaneously allows relatively uniform doping of large area graphene that is transferred onto the Si membrane^21^. Here, the TTE structure was developed on PI via conventional photolithography and metal patterning (~100 nm Au and ~10 nm Ti) and printed with a flat PDMS stamp rather than the micro-tip stamp (see Supplementary Fig. 19). Figure 5b–d confirm that the stacked MoSM/GoSM can be printed onto the bottom TTE layer, despite the bumpy surface. During the GoSM fabrication, the sidewall of the Si membrane was covered with graphene (i.e., when transferring graphene onto the suspended Si membrane, as described in Supplementary Fig. 3). Therefore, the graphene on the Si membrane was in direct contact with the TTE (see Supplementary Fig. 20). Double modular transfer printing enabled the direct matching between the ‘I’ beam metamaterial and graphene (i.e., the transfer of MoSM from the mother substrate to the temporal mediate layer with a low adhesion (PDMS with a 2:1 base to curing ratio), followed by the retrieval of the MoSM from temporal mediate layer and the subsequent modular printing of MoSM onto GoSM). The printing of the top TTE layer, which was assisted by the epoxy layer (Norland Optical Adhesive, NOA 61), finished the entire construction of electrically-gated graphene metadevice (Fig. 5e).

The electrical modulation of the THz amplitude transmittance was measured with the constructed graphene devices. The upper limits of the applied gate voltage for both the graphene-only device and the graphene metamaterial device were approximately 150 V, beyond which the dielectric breakdown occurred. The gate voltage-dependent transmission characteristics for both devices are shown in Fig. 5f,g. From these plots, it is clear that the 3D heterogeneous modular assembly can be applied to the construction of active metadevices. Therefore, the modular printing strategy may allow us to realize functional metadevices in a versatile way and to expand the range of possible architectures of metadevices.

## Discussion

The 3D modular transfer printing was successfully utilized to construct 2D/3D metamaterials and metadevices in which a wide range of dissimilar materials/substrates and different levels of structural features were heterogeneously integrated. In particular, the use of non-specific and dry adhesion with extremely high switchability makes the modular transfer printing strategy proposed here readily adaptable to the hybridization between broad classes of dissimilar materials and substrates as well as with the 3D integration of different meta-atoms. Additionally, the modular transfer printing of each distinct meta-atom and functional material described here allows the rapid construction of sophisticated 2D/3D metamaterials and metadevices with desired photonic signatures and tunability. Thus, this method may provide the platform for the heterogeneous 2D/3D assembly of metamaterials and metadevices with a wide range of material accessibility for the expansion of their possible applications in relevant photonic technologies.

## Methods

### Fabrication of MoSM and GoSM

All of the MoSM and GoSM were developed using a collective set of monolithic microfabrication processes, including photolithographic patterning of photoresist (PR), metal evaporation (e-beam evaporation), metal lift-off, and undercut etching.

Regarding the MoSM fabrication (also summarized in Supplementary Fig. 2), the designed THz metamaterial was first developed on a silicon-on-insulator (SOI) wafer (Ultrasil Corp.) via photolithography and successive Au lift-off (see Supplementary Fig. 2a,b). Here, the footprint of the THz metamaterial was carefully chosen to match the lateral dimension of the Si membrane. The thickness of the top Si layer was 2 or 3 μm. The lateral dimension of the Si membrane was defined by the photolithographic patterning of PR (AZ5214 from MicroChem, 1 μm thick) and successive reactive ion etching (RIE; Plasma-Them), as summarized in Supplementary Fig. 2c,d. Then, we partially etched the buried silica box layer (300 nm thickness) by dipping the wafer into hydrofluoric acid (HF) (see Supplementary Fig. 2f,g). The etched lateral dimension of silica box layer was controlled to be 200 nm. For this process, the dipping time was carefully adjusted to create an appropriate undercut trench below the borders of the defined MoSM (see Supplementary Fig. 2g). To define the PR anchor, we coated the overall wafer with PR (AZ5214) and performed flood exposure using ultraviolet (UV) light at a wavelength of 365 nm (see Supplementary Fig. 2h). In this case, the borders of the top MoSM acted as a mask; thus, the PR (AZ5214) below the borders of the defined MoSM were retained after the wet-development process using a basic developer (AZ 327 MIF) (see Supplementary Fig. 2i). We also formed an ‘HF entrance’ to facilitate the undercut etching of the buried silica box layer (see Supplementary Fig. 2j). The photomask, which was used in the process of defining the Si membrane plate, was carefully aligned to be partially overlapped with the already formed MoSM (rotation with 15 degrees). Then, the PR was over-exposed with UV. The over-exposure of UV light can lead to the photoreaction of the PR even below the borders of the MoSM; therefore, a wet treatment with a basic developer resulted in the etching of the PR (as shown in Supplementary Fig. 2k). Then, the buried silica box layer was fully etched via HF treatment.

Regarding the GoSM fabrication (also summarized in Supplementary Fig. 3), we first developed an array of Si membranes, which were suspended onto PR anchors. This process was exactly the same as the fabrication of MoSM except for the THz metamaterial patterning onto a SOI wafer. Then, graphene, which was grown via chemical vapour deposition (CVD) on a copper film (Graphene Square, Inc.), was transferred to the Si membrane array (see Supplementary Fig. 3a,b). The photolithographic patterning of PR (AZ5214) and the successive RIE defined lateral geometry of the graphene corresponded to that of the Si membranes (see Supplementary Fig. 3c). Finally, a polymeric support (polymethylmethacrylate (PMMA), MicroChem) that was used in the graphene transfer process was carefully removed (see Supplementary Fig. 3d).

### Fabrication of the elastomeric microtip stamp

The master pattern of the PDMS 5-microtip stamp was developed on a Si (100) wafer (Addison Engineering). First, 100-nm-thick silicon nitride (Si_x_N_y_) was deposited onto a Si (100) wafer via plasma-enhanced CVD (PECVD). then, four 100 μm × 100 μm square holes (at the corners) and one 150 μm × 150 μm square hole (at the centre) were patterned onto Si_x_N_y_ via photolithographic patterning of PR and successive HF wet etching. This square hole-patterned Si_x_N_y_/Si (100) wafer was dipped into KOH solution, and the temperature of the KOH solution was increased to 80 °C. The punched Si_x_N_y_ acted as a hard mask for the anisotropic etching of the Si (100). The opened Si (100) was selectively etched to form 5 pyramidal pits. After the removal of the Si_x_N_y_ hard mask via HF etching, 5 pyramidal pits (four 100 μm × 100 μm squares at the corners; one 150 μm × 150 μm square at the centre) were obtained on the Si (100) wafer. The epoxy resin (550 μm-thick SU8 from MicroChem) was coated onto the prepared Si (100) wafer, and we performed photolithography to define the template of the elastomeric stamp post. This developed master pattern was treated with trichlorosilane (United Chemical Technology) to reduce the surface energy. Finally, we casted the prepolymer of PDMS (Sylgard184, Dow Corning; a 5:1 mixture of base to curing agent) onto the prepared master and cured it. The thickness of the backing layer of PDMS was adjusted to approximately 2 mm. Peeling the cured PDMS stamp completed the 5-microtip stamp preparation process.

### 3D modular transfer printing

All of the results, except those presented in Fig. 2c, were obtained using custom-built translational stages (shown in Fig. S11). One *x*-, *y*-, *z*-axis translational stage for the stamp and another *x*-, *y*-, *z*-axis translational stage with rotational controllability were independently manipulated. A vision optical system was also equipped to monitor the adhesion-on and -off states and the spatial position of the stamp and MoSM/GoSM. After the 3D modular transfer printing of MoSM or GoSM, thermal annealing (330 °C for 2 hours) or PI encapsulation (PI-2610, HD MicroSystems) was performed to complete the fabrication of the metamaterials and metadevices. To demonstrate large-area fabrication, we also used an automated transfer printing system (see Fig. S12).

### THz measurement

We performed THz time-domain spectroscopy (THz-TDS) to investigate the THz properties of the printed 2D/3D metamaterials and metadevices. We used a custom-built THz-TDS system that was encapsulated in a custom-built box. The measurements were all conducted in an environment that was purged with nitrogen gas (relative humidity was below ~1.0%). A high voltage supply (Keithley 2400 SourceMeter^®^) was used to characterize the gate-voltage-dependent THz transmittance through the 3D modular transfer printed metadevices, and this voltage supply was connected to the bottom (graphene connected) and top TTE layers.

### Numerical simulations

To theoretically predict the THz properties of the constructed 3D metamaterials and metadevices, we performed finite element analysis with a commercially available software package (i.e., CST Microwave Studio). The complex permittivity of gold at the frequency of interest was taken from the literature^31^, whereas those values for HRFZ Si, Pi, PDMS, and NOA were measured using our THz-TDS system. We also conducted finite element analysis of the stain distribution of the bended Si membrane/PI composite using commercially available COMSOL Multiphysics software.

### Adhesion test

To measure the adhesion force between the elastomeric 5-microtip stamp and the Si surface, we used a custom testing setup, as shown in our previous works^26^. Here, a clean, smooth, and flat Si disk was directly connected to a precision load cell (transducer Techniques, GSO-10, ~50 μN resolution). The pulling speed was precisely controlled using an automated vertical translational stage (Aerotech, PRO165, 500 nm resolution).

## Additional Information

**How to cite this article**: Lee, S. *et al*. Heterogeneously Assembled Metamaterials and Metadevices via 3D Modular Transfer Printing. *Sci. Rep*. **6**, 27621; doi: 10.1038/srep27621 (2016).

## Supplementary Material

Supplementary Information

## References

[b1] Veselago V. G. The electrodynamics of substrances with simultaneously negative values of ε and μ. Sov. Phys. Usp. 10, 509–514 (1968).

[b2] Smith D. R., Padilla J. W., Vier D. C., Nemat-Nasser S. C. & Schultz S. Compostie medium with simultaneously negative permeability and permittivity. Phys. Rev. Lett. 84, 4184–4187 (2000). 10990641 10.1103/PhysRevLett.84.4184

[b3] Pendry J. B. Negative refraction makes a perfect lens. Phys. Rev. Lett. 85, 3966–3969 (2000). 11041972 10.1103/PhysRevLett.85.3966

[b4] Shelby R. A., Smith D. R. & Schultz S. Experimental verification of a negative index of refraction. Science 292, 77–79 (2001). 11292865 10.1126/science.1058847

[b5] Eleftheriades G. V., Lyer A. K. & Kremer P. C. Planar negative refractive index media using periodically L-C loaded transmission lines. *IEEE Trans*. Microwave Theory Tech. 50, 2702–2712 (2002).

[b6] Pendry J. B., Schrurig D. E. & Smith D. R. Controlling electromagnetic fields. Science 312, 1780–1782 (2006). 16728597 10.1126/science.1125907

[b7] Schurig D. . Metamaterial electromagnetic cloak at microwave frequencies. Science 314, 977–980 (2006). 17053110 10.1126/science.1133628

[b8] Cai W. S., Chettiar U. K., Kildishev A. V. & Chalaev V. M. Optical cloaking with metamaterials. Nature Photon. 1, 224–227 (2007).

[b9] Valentine J. . Three-dimensional optical metamaterial with a negative refractive index. Nature 455, 376–379 (2008). 18690249 10.1038/nature07247

[b10] Liu R. . Experimental demonstration of electromagnetic tunneling through an epsilon-near-zero metamaterial at microwave frequencies. Phys. Rev. Lett. 100, 023903 (2008). 18232869 10.1103/PhysRevLett.100.023903

[b11] Zhang S. . Negative refractive index in chiral metamaterials. Phys. Rev. Lett. 102, 023901 (2009). 19257274 10.1103/PhysRevLett.102.023901

[b12] Choi M. . A terahertz metamaterial with unnaturally high refractive index. Nature 470, 369–373 (2009). 21331038 10.1038/nature09776

[b13] Morita P. . Realization of an all-dielectric zero-index optical metamaterial. Nature Photon. 7, 791–795 (2013).

[b14] Rogacheva A. V., Fedotov V. A., Schwanecke A. S. & Zheludev N. I. Giant gyrotropy due to electromagnetic-field coupoing in a bilayered chiral structure. Phys. Rev. Lett. 97, 177401 (2006). 17155505 10.1103/PhysRevLett.97.177401

[b15] Kim T.-T. . Optical activity enhanced by strong inter-molecular coupling in planar chiral metamaterials. Sci. Rep. 4, 5864 (2014). 25209452 10.1038/srep05864 PMC4160707

[b16] Yu N. . Light propagation with phase discontinuities: generalized laws of relfection and refraction. Science 334, 333–337 (2011). 21885733 10.1126/science.1210713

[b17] Lin D., Fan P., Hasman E. & Brongersma M. L. Dielectric gradient metasurface optical elements. Science 345, 298–302 (2014). 25035488 10.1126/science.1253213

[b18] Chen H. T. . Active terahertz metamaterial devices. Nature 444, 597–600 (2006). 17136089 10.1038/nature05343

[b19] Chen H. T. . A metamaterial solid-state terahertz phase modulator. Nature Photon. 3, 148–151 (2009).

[b20] Hu T., Padilla W. J., Xin Z. & Averitt R. D. Recent progress in electromagnetic metamaterial devices for terahertz applications. IEEE J. Sel. Top. Quan. Electron. 17, 92–101 (2011).

[b21] Lee S. H. . Switching terahertz waves with gate-controlled active graphene metamaterials. Nature Mater. 11, 936–941 (2012). 23023552 10.1038/nmat3433

[b22] Yao Y. . Electrically tunable metasurface perfect absorbers for ultrathin mid-infrared optical modulator. Nano Lett. 14, 6526–6532 (2014). 25310847 10.1021/nl503104n

[b23] Mousavi S. H. . Inductive tuning of Fano-resonant metasurfaces using plasmonic response of graphene in the mid-infrared. Nano Lett. 13, 1111–1117 (2013). 23398172 10.1021/nl304476b

[b24] Chanda D. . Large-area flexible 3D optical negative index metamaterial formed by nanotransfer printing. Nature Nanotechnol. 6, 402–407 (2011). 21642984 10.1038/nnano.2011.82

[b25] Meitl M. A. . Transfer printing by kinetic control of adhesion to an elastomeric stamp. Nature Mater. 5, 33–38 (2006).

[b26] Kim S. . Microstructured elastomeric surfaces with reversible adhesion and examples of their use in deterministic assembly by transfer printing. Proc. Natl. Acad. Sci. USA 107, 17095–17100 (2010). 20858729 10.1073/pnas.1005828107 PMC2951455

[b27] Keum H. . Silicon micro-masonry using elastomeric stamps for three-dimensional microfabrication. J. Micromech. Microeng. 22, 055018 (2012).

[b28] Lee S. . Reversibly stretchable and tunable terahertz metamaterials with wrinkled layouts. Adv. Mater. 24, 3491–3497 (2012). 22688807 10.1002/adma.201200419

[b29] Tao H. . Silk-based conformal, adhesive, edible food sensors. Adv. Mater. 24, 1067–1072 (2012). 22266768 10.1002/adma.201103814

[b30] Kim S. . Imbrcate scales as a design construct for microsystems technologies. Small 8, 901–906 (2012). 22180159 10.1002/smll.201101832

[b31] Ordal M. A. . Optical properties of the metals Al, Co, Cu, Au, Fe, Pb, Ni, Pd, Pt, Ag, Ti, and W in the infrared and far infrared. Appl. Opt. 22, 1099–1119 (1983). 18195926 10.1364/ao.22.001099

