# Supplementary material for: Heterogeneously Assembled Metamaterials and Metadevices via 3D Modular Transfer Printing
Source: Sci Rep. 2016 Jun 10;6:27621. doi: 10.1038/srep27621 (PMC4901332; doi:10.1038/srep27621)
Supplement: Supplementary Information [file srep27621-s1.pdf]

Supplementary Information for "Heterogeneously Assembled Metamaterials and Metadevices via 3D Modular Transfer Printing"

Seungwoo Lee[1], Byungsoo Kang[2], Hohyun Keum[3], Numair Ahmed[3], John A. Rogers[4], Placid M. Ferreira[3], Seok Kim[3], and Bumki Min[2]

[1] *SKKU Advanced Institute of Nanotechnology (SAINT) & School of Chemical Engineering, Sungkyunkwan University (SKKU), Suwon 16419, Republic of Korea*

[2] *Department of Mechanical Engineering, Korea Advanced Institute of Science and Technology (KAIST), Daejeon 34141, Republic of Korea*

[3] *Department of Mechanical Science and Engineering, University of Illinois, Urbana-Champaign, Illinois 61801, USA*

[4] *Department of Materials Science and Engineering, University of Illinois, Urbana-Champaign, Illinois 61801, USA*

Here, we present detailed information on the 3D modular transfer printing-enabled construction of heterogeneously assembled metamaterials and metadevices, including (i) the preparation and characterization of MoSM and GoSM building blocks, (ii) the design and adhesion measurement of the elastomeric 5-microtip stamp for a high level of adhesion switching, (iii) snapshots of the 3D modular transfer printing of MoSM, (iv) the capability for 3D modular transfer printing on universal substrates, (v) the manual and automated transfer printing machines, (vi) the food sensor and stretchable metamaterial

platform, (vii) the alignment error of the 3D printing of MoSM, (viii) the encapsulated 3D metamaterials, and (ix) the graphene metadevices.

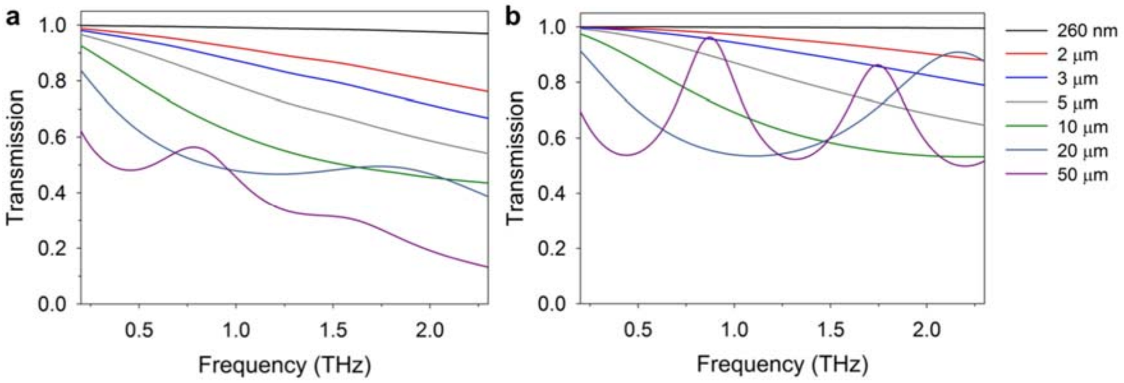

Supplementary Figure 1: Simulated THz amplitude transmittance of Silicon (Si) at varying thicknesses (**a**) *p*-doped Si with an Im (ε) of 2.7, (**b**) High Resistivity Float Zone (HRFZ) Si with an Im (ε) of 0.4.

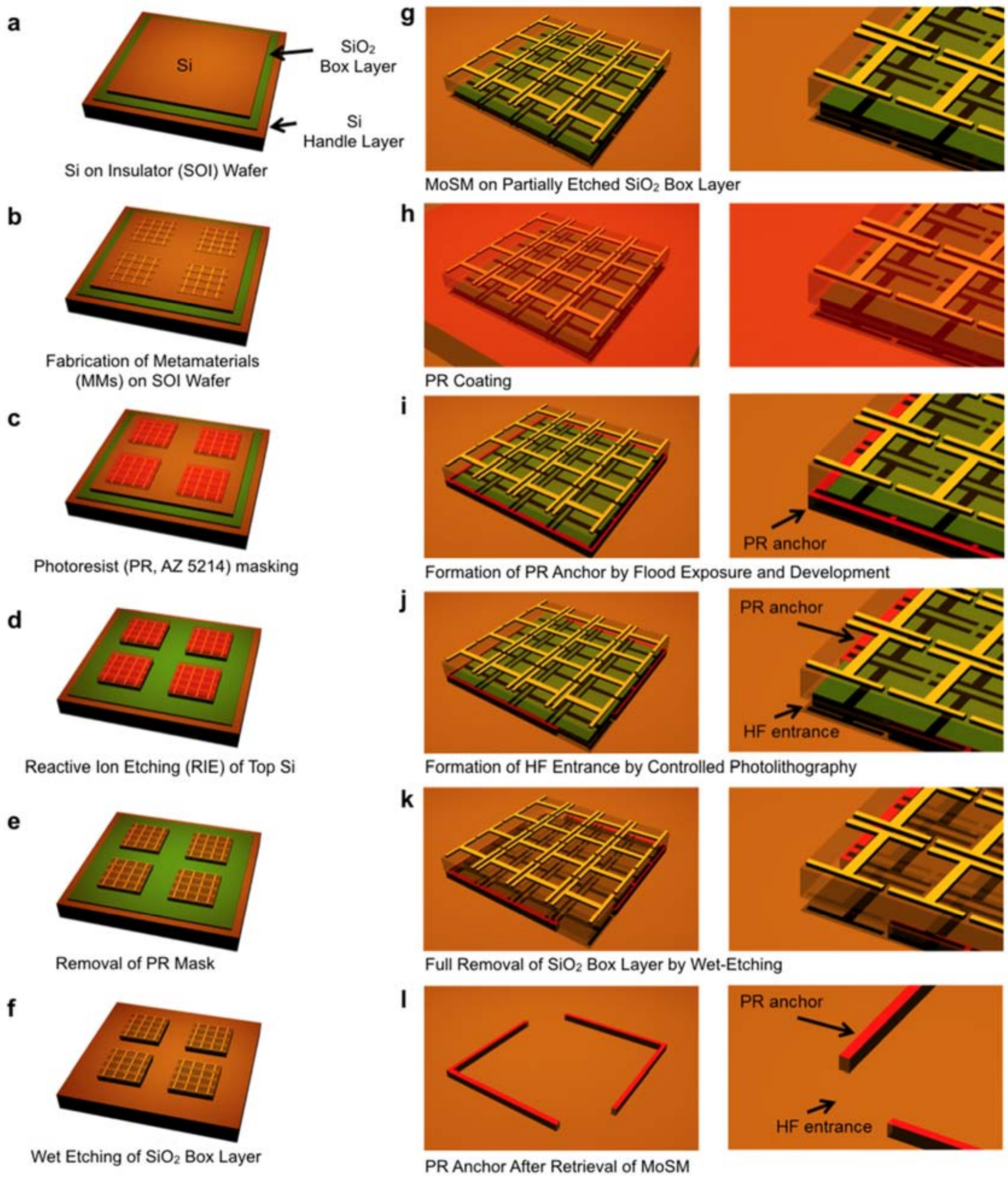

Supplementary Figure 2: **Fabrication of a Metamaterial on a Si Membrane (MoSM)** Schematic overview of the fabrication of MoSM in a suspended form onto a photoresist (PR) anchor.

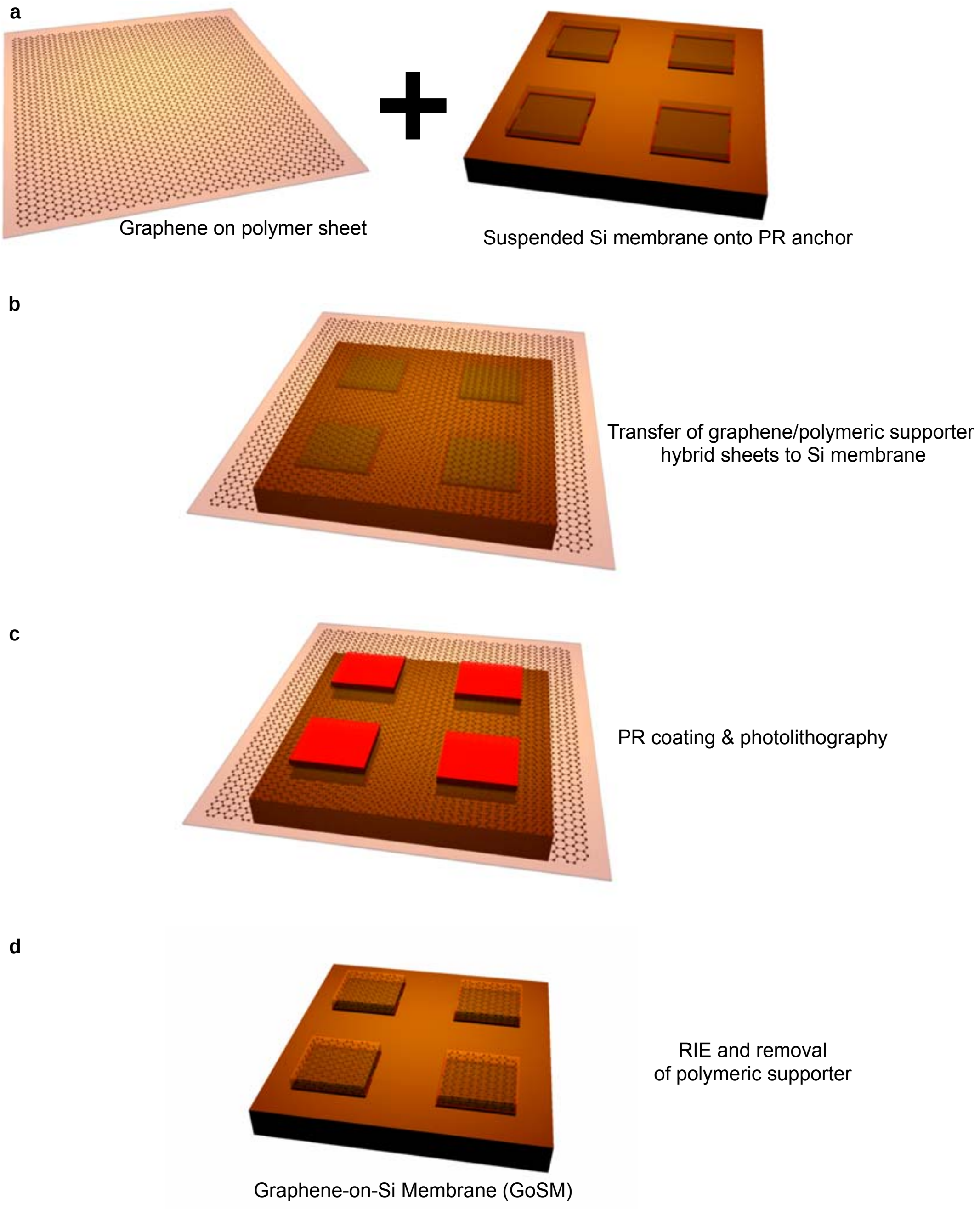

Supplementary Figure 3: **Fabrication of Graphene on a Si Membrane (GoSM)** Schematic overview of the fabrication of GoSM in a suspended form onto a photoresist (PR) anchor. Here, polymethylmethacrylate (PMMA) was used as the polymeric supporter for the graphene transfer.

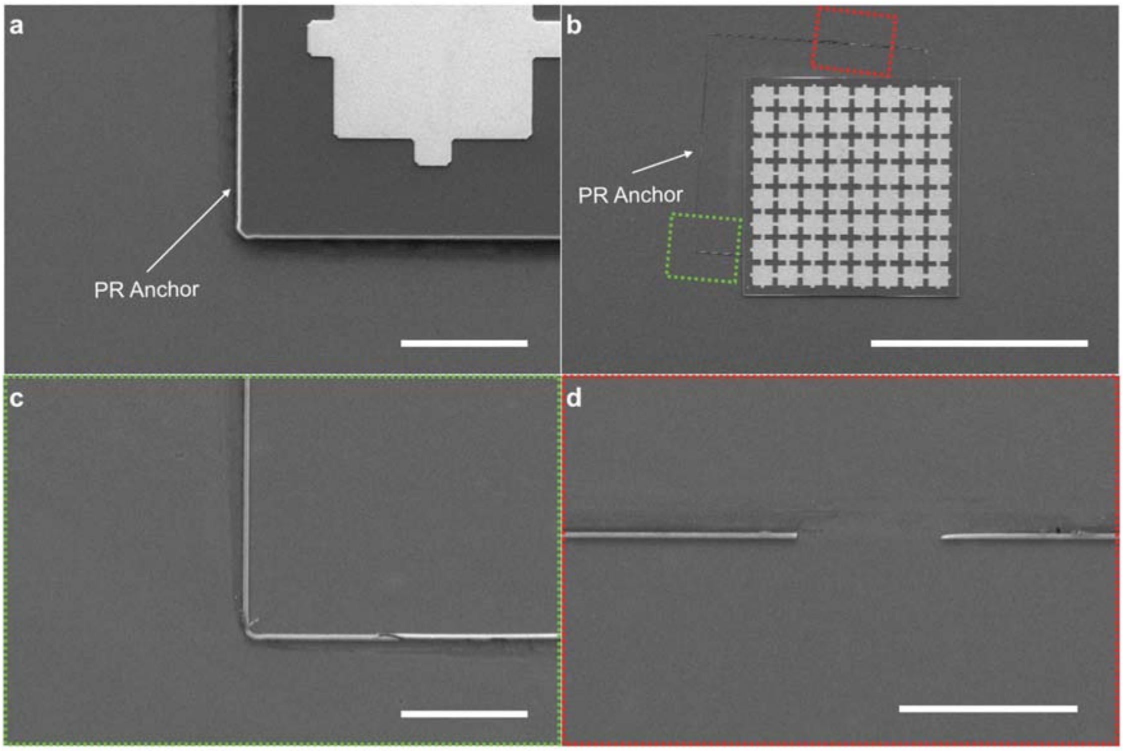

Supplementary Figure 4: **Structural features of the MoSM (or GoSM) suspended on PR anchors** (**a**) Scanning electron microscope (SEM) image of the as-prepared MoSM on PR anchors (i.e., self-aligned geometry). The scale bar is 35 μm. (**b**) Misaligned MoSM and PR anchors (after retraction of the MoSM and subsequent printing of the MoSM onto the PR anchors). The scale bar is 600 μm. (**c**) Highly-magnified SEM image of the PR anchors. The scale bar is 10 μm. (**d**) Highly-magnified SEM image of the HF entrance. The scale bar is 10 μm.

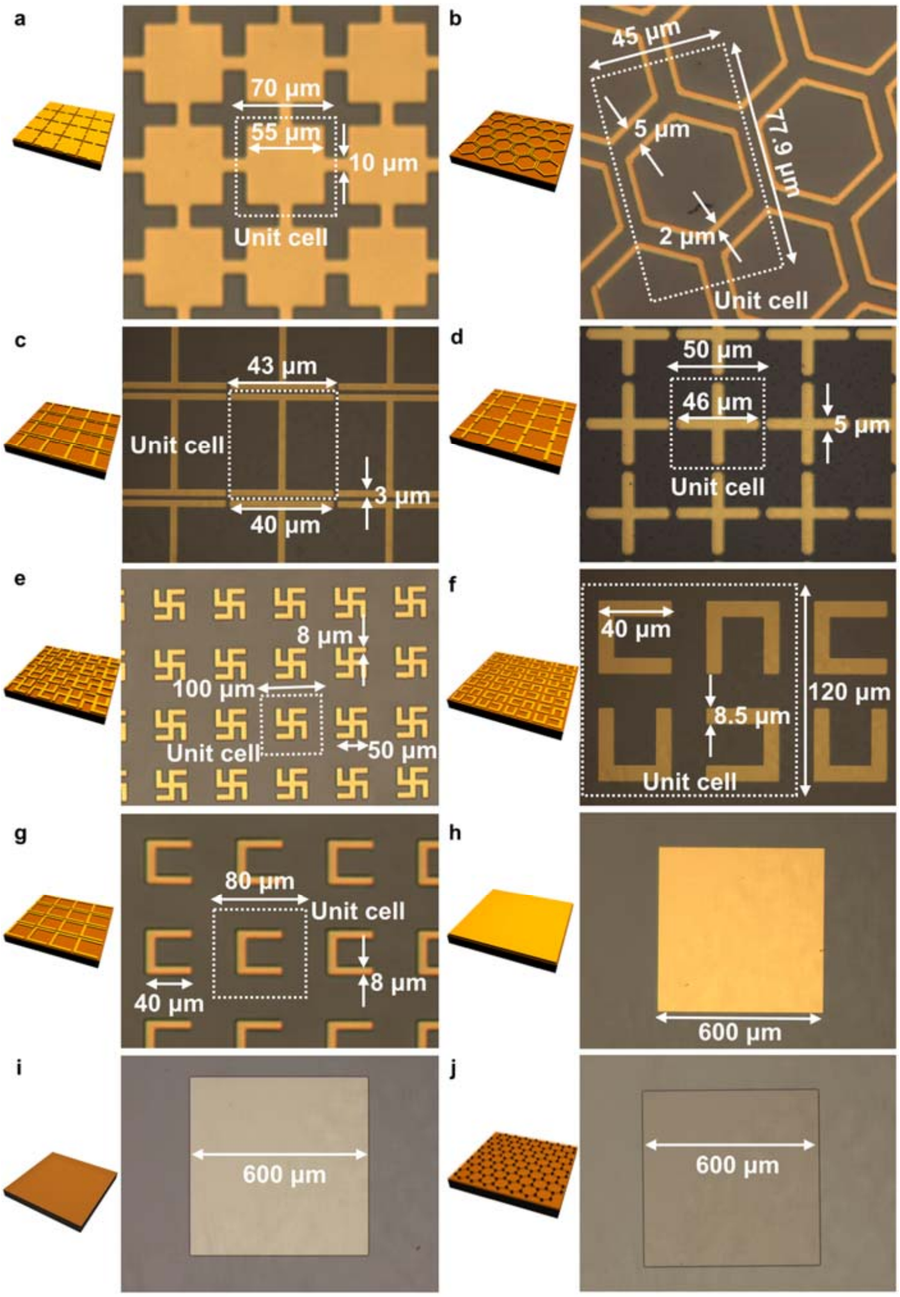

Supplementary Figure 5: Family of building blocks used in this work (top-view optical microscope (OM) images) (**a**) fishnet MoSM, (**b**) honeycomb MoSM, (**c**) ‘I’ beam MoSM, (**d**) cross MoSM, (**e**) gammadion MoSM, (**f**) 90° twisted ‘U’ resonator MoSM, (**g**) ‘U’ resonator MoSM, (**h**) flat Au/Si membrane, (**i**) Si membrane, and (**j**) GoSM.

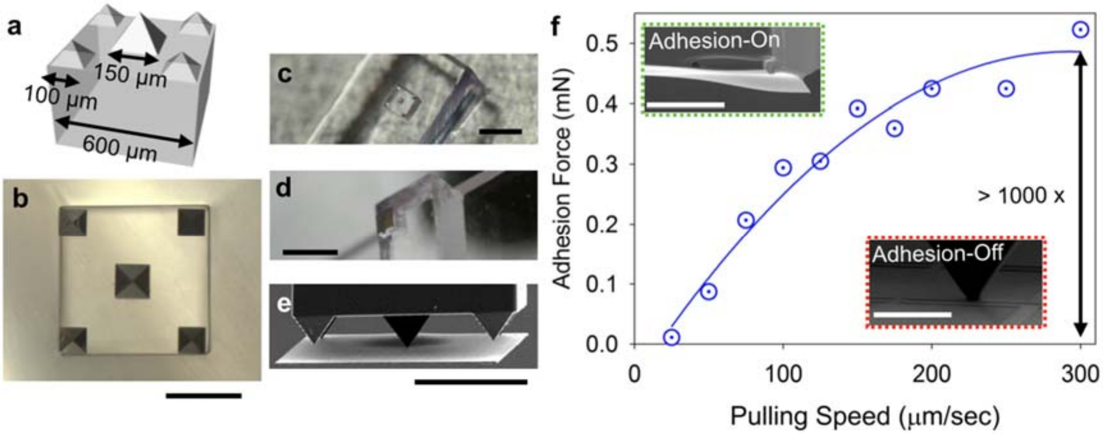

Supplementary Figure 6: **Elastomeric 5-microtip stamp with extremely high adhesion switchability** (**a**) Schematic of an elastomeric 5-microtip stamp with detailed dimensional parameters. The height of the fabricated stamp was 550 μm. (**b**) Top view OM image of the 5-microtip stamp. The scale bar is 300 μm. (**c**) Image of the 5-microtip stamp attached onto slide glass (1 mm thickness). The scale bar is 1.2 mm. (**d**) Macroscopic image of the 5-microtip stamp inked with MoSM. The scale bar is 1.2 mm. (**e**) Side-view SEM image of the 5-microtip stamp inked with MoSM (adhesion-off state). The scale bar is 300 μm. (**f**) Measured adhesion force between the 5-microtip stamp and the flat Si surface as a function of the pulling speed. Insets show SEM images of the 5-microtip stamp with adhesion-on and –off states. All scale bars are 100 μm.

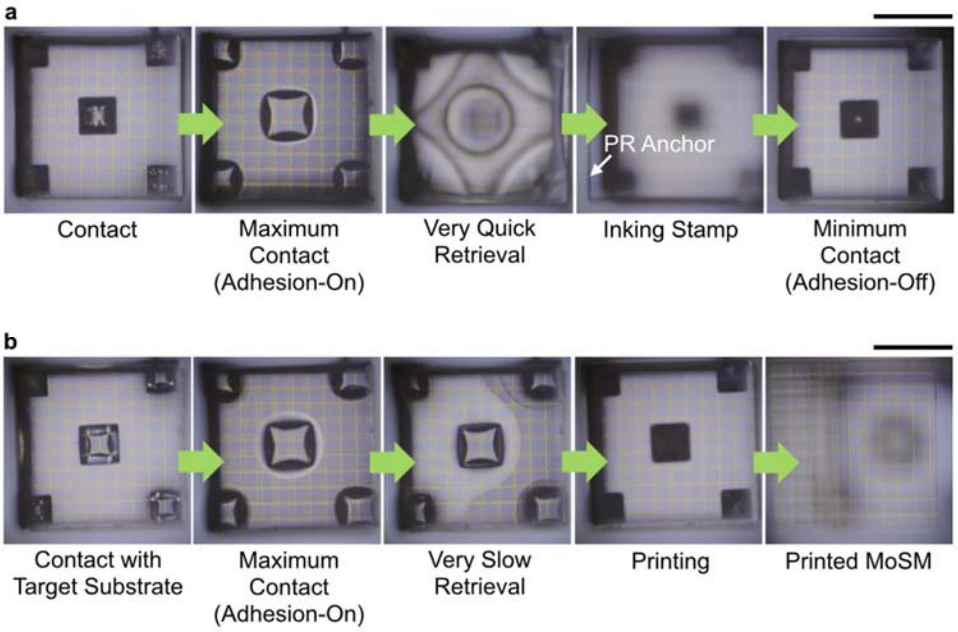

Supplementary Figure 7: Snapshots (OM images) of 3D modular transfer printing (**a**) Retraction of MoSM (‘I’ beam metamaterials) using an elastomeric 5-microtip stamp. (**b**) Printing of MoSM. All scale bars are 300 μm.

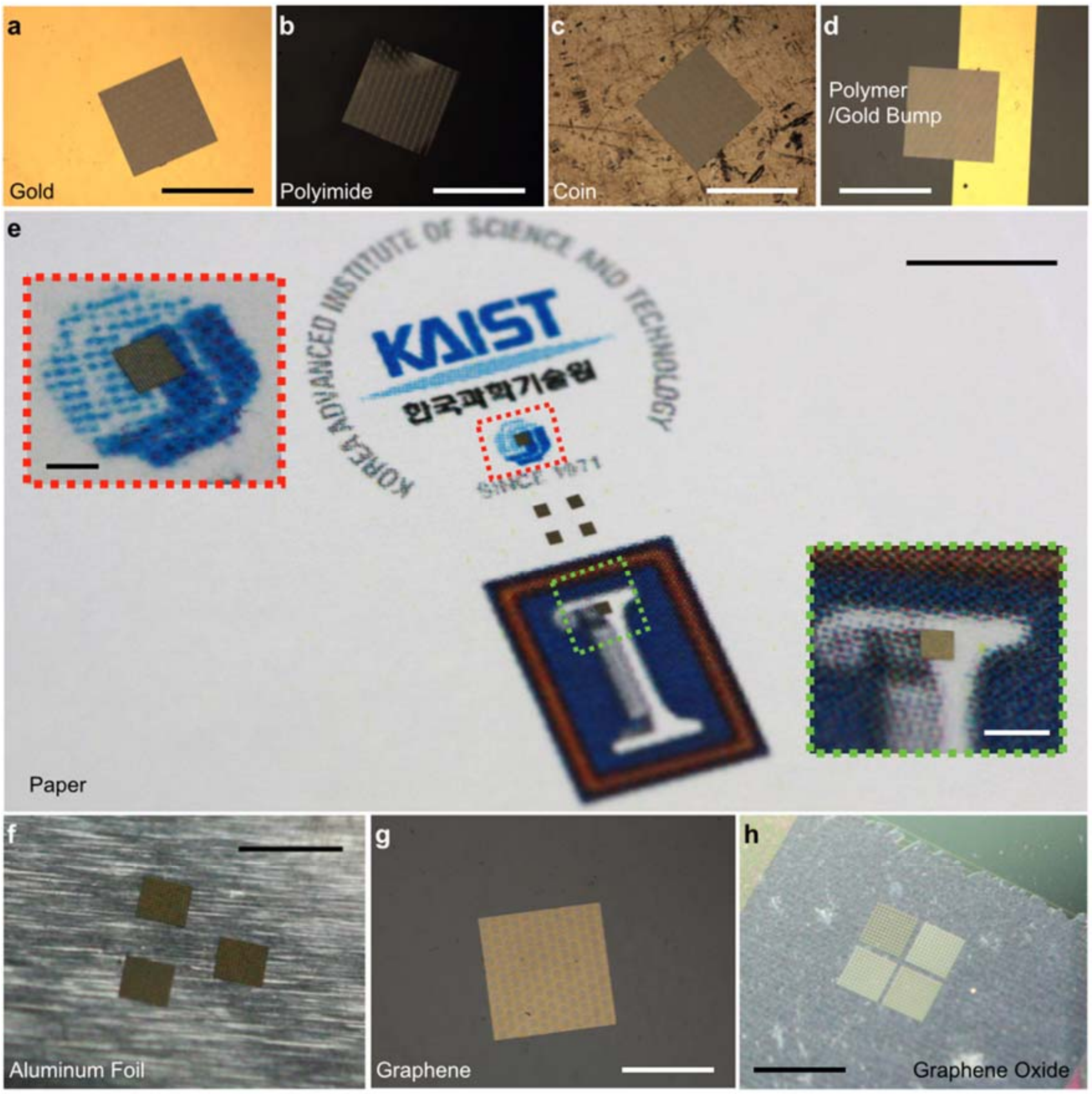

Supplementary Figure 8: **3D modular transfer printing of MoSM onto universal substrates** (**a**) Flat gold, (**b**) Flat polyimide (PI) film, (**c**) Coin (rough metal), (**d**) 100 nm-thick gold strip on PI film (substrate with two-level height). All scale bars are 600 μm. (**e**) Commercially available paper (porous and rough carbon fibre mesh). The scale bar is 8 mm, and the scale bars in the red and green dotted boxes are 600 μm and 1.5 mm, respectively. (**f**) Aluminium foil (rough metal). The scale bar is 1.5 mm. (**g**) Graphene. The scale bar is 600 μm. (**h**) Stacked graphene oxide (GO) paper. The scale bar is 1.3 mm.

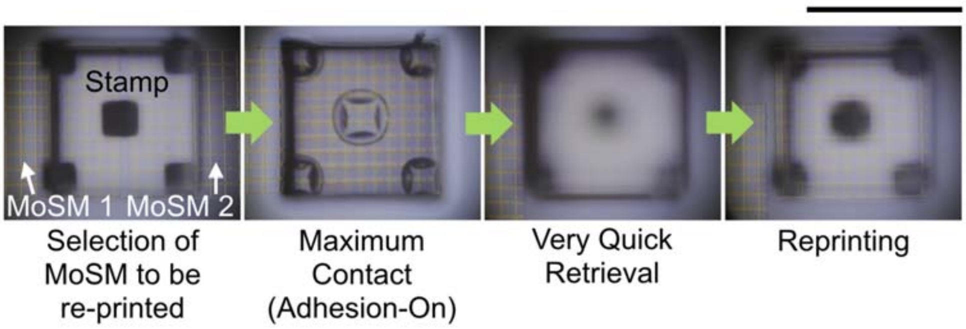

Supplementary Figure 9: **Reversibility of 3D modular transfer printing** The printed MoSM can be retracted and re-printed repeatedly. The scale bar is 600 μm.

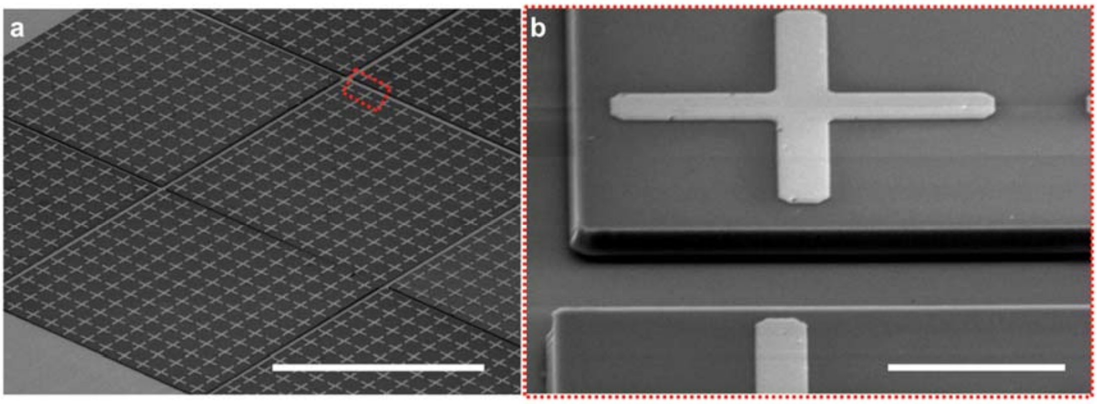

Supplementary Figure 10: **Uniformity of the printed MoSMs** (**a**) Low-magnification SEM image of the printed cross MoSM array on a PI substrate. The scale bar is 600 μm. (**b**) Magnified SEM image of the red dotted box in (**a**), which shows the small misalignment between the printed MoSMs. The scale bar is 20 μm.

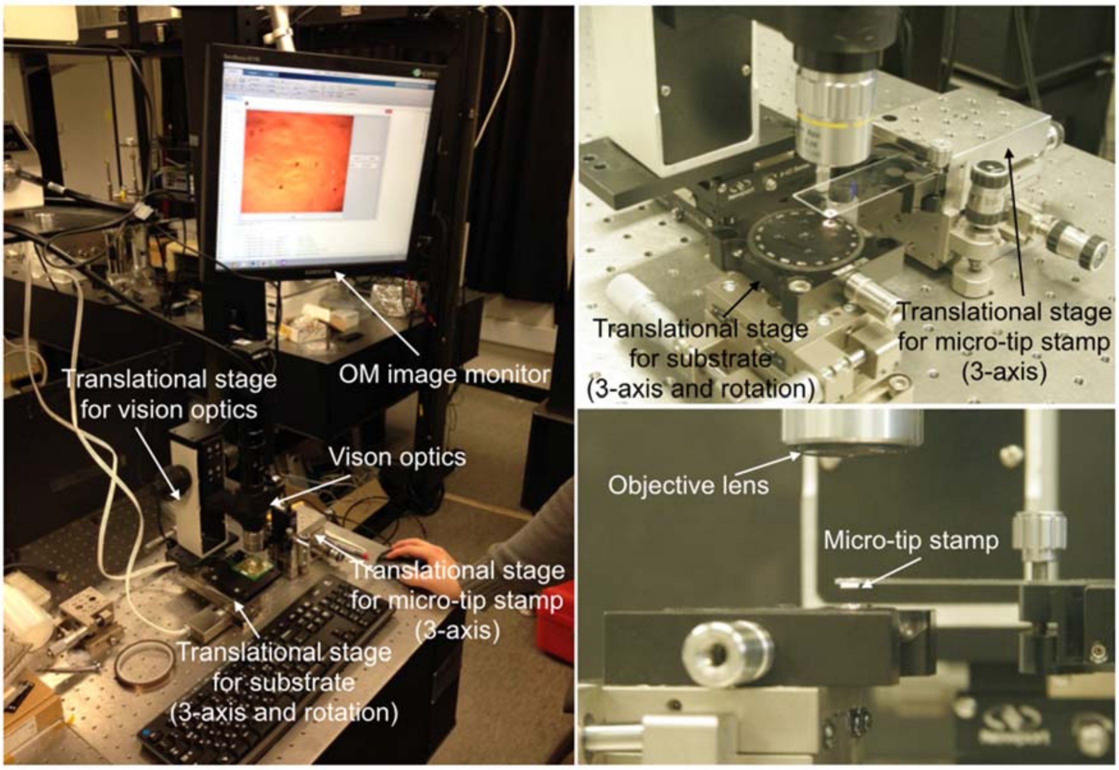

Supplementary Figure 11: Custom-built manual transfer printing machine that was used in this work.

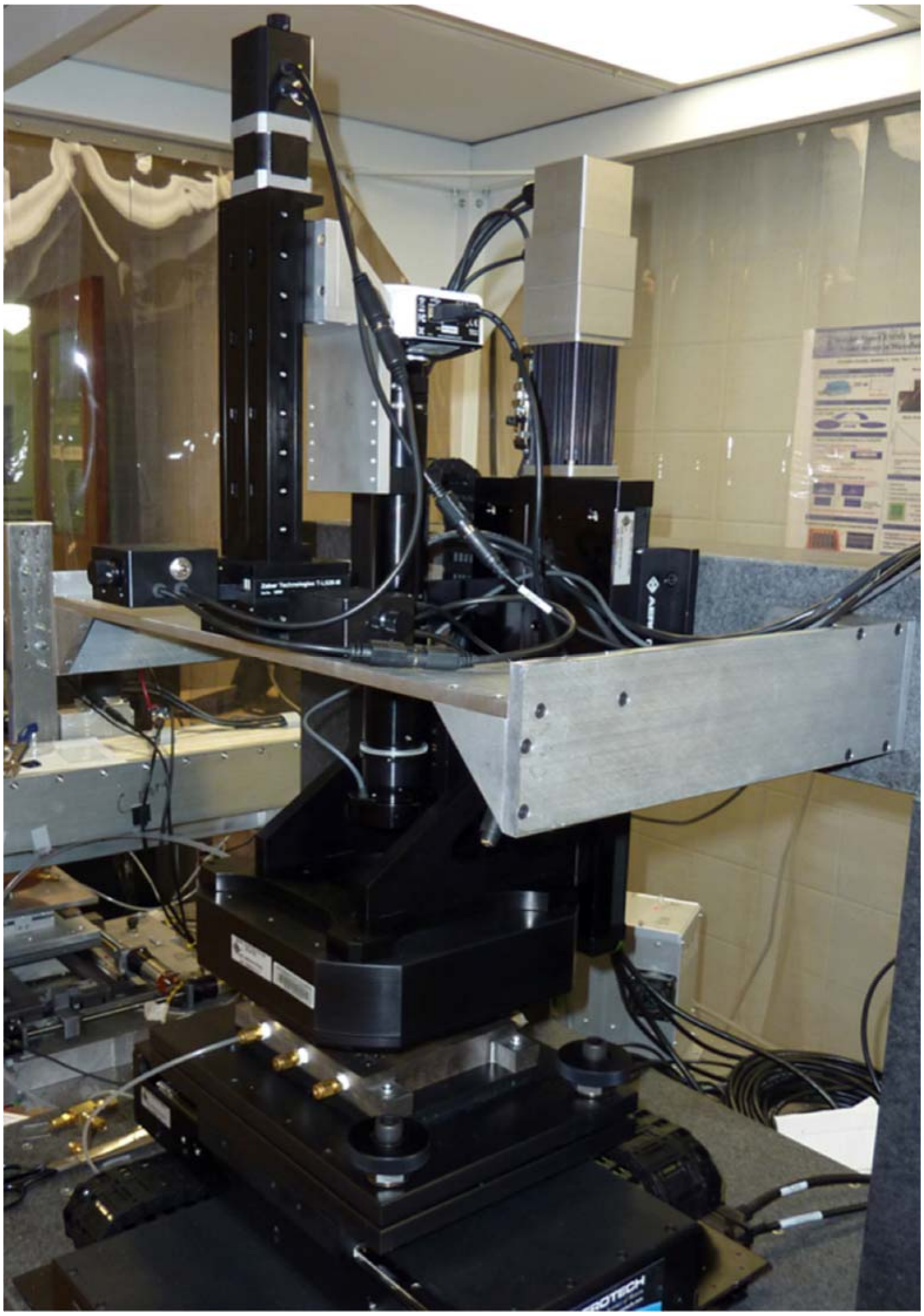

Supplementary Figure S12: **Automated transfer printing machine.**

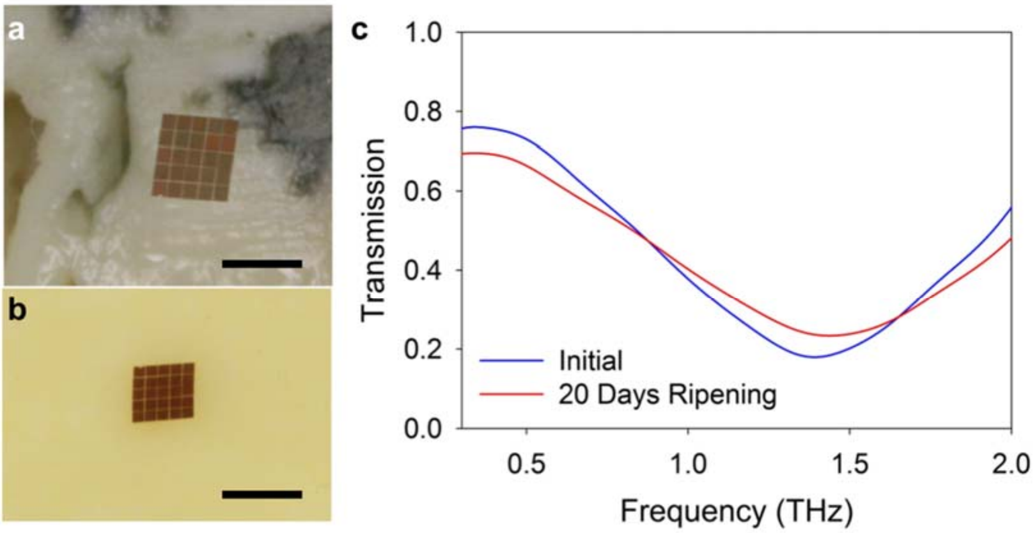

Supplementary Figure 13: Remote metamaterial antenna for the food sensor (**a**) 5 x 5 honeycomb MoSM array that was printed on stilton cheese, (**b**) 5 x 5 honeycomb MoSM array that was printed on cheddar cheese. The scale bars are 3.5 mm for (**a**) and 4.0 mm for (**b**). (**c**) Experimentally measured change in the THz amplitude transmittance of the 5 x 5 honeycomb MoSM that was printed on stilton cheese during ripening. For the THz measurement, bulk cheese was sliced into a 2 μm thick layer using a doctor blade. Then, the 5 x 5 honeycomb MoSM was printed onto the thin cheese layer.

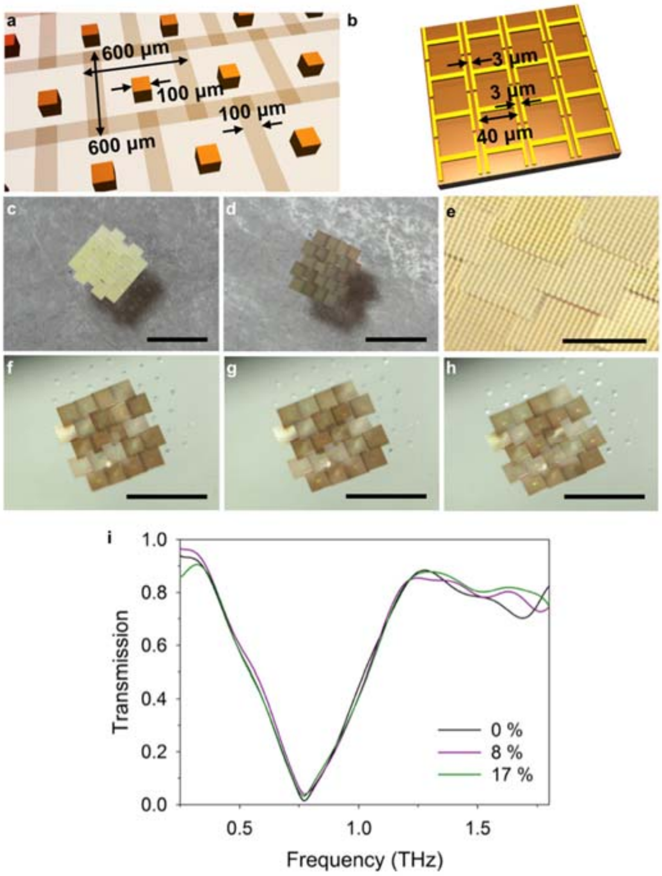

Supplementary Figure 14: **Stretchable THz metamaterials with an imbricate geometry** (**a**) Schematic for the stretchable Si membrane (or MoSM) array with an imbricate layout. Herein, each MoSM was designed to be docked onto the PDMS square post array with a 100 μm lateral dimension and a 50 μm height. Toward this direction, the MoSM was chemically linked to the PDMS square post via a hydroxyl condensation reaction. Each MoSM was overlapped by a 100 μm lateral dimension. (**b**) Schematic of MoSM ('I' beam metamaterials) used for constructing the stretchable metamaterial with the imbricate layout. (**c-e**) Printed and stretchable THz metamaterials with imbricate geometry. The scale bars in (**c-d**) and (**e**) are 2.6 mm and 600 μm, respectively. Macroscopic images of the stretched metamaterials with a (**f**) 0 %, (**g**) 8 %, and (**h**) 17 % stretching ratio. Until the 17 % stretched state, the metamaterial retained 100 % of the areal coverage because of the imbricate layout. All scale bars are 2.0 mm. (**i**) THz amplitude transmittance of the stretchable 'I' beam metamaterials with different stretching ratios. Due to the imbricate geometry, the THz response of the metamaterial remained unchanged even at a stretching ration of 17 %.

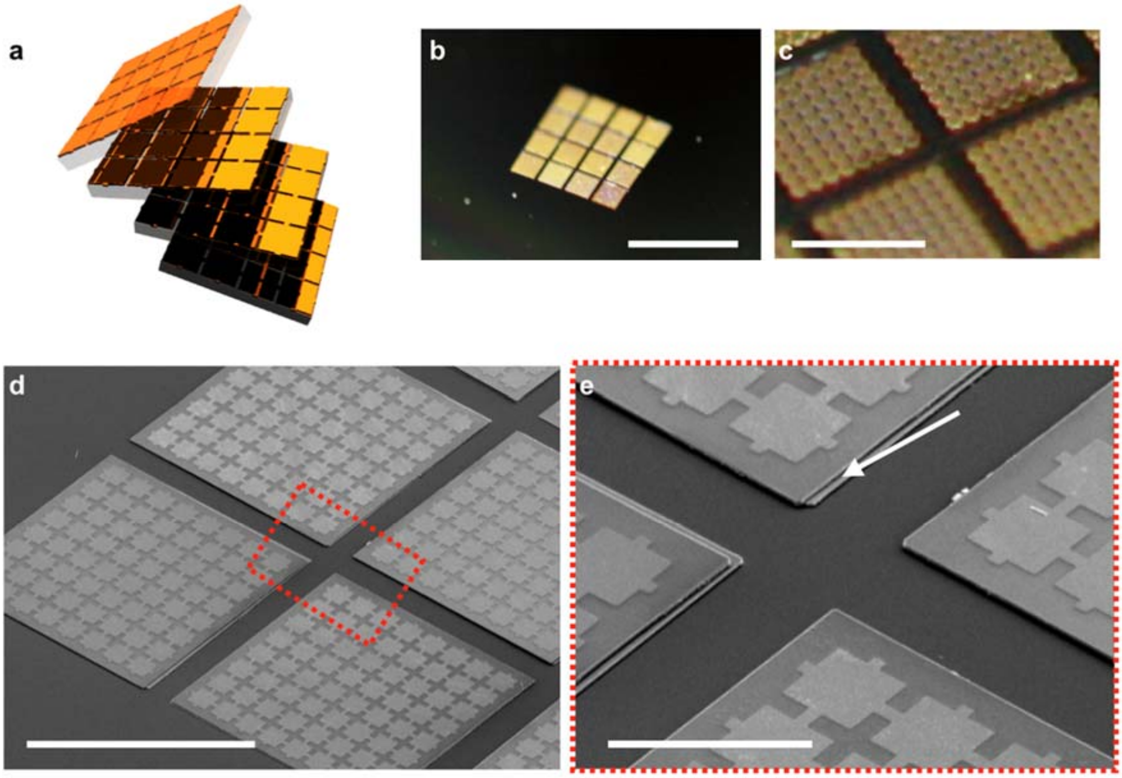

Supplementary Figure 15: **Alignment accuracy of the 3D modular transfer printing** (**a**) Schematic of the 3D stacked fishnet MoSMs, (**b**) Image of the 2-layered fishnet MoSM array (4 x 4). The scale bar is 2.5 mm. (**c**) High-magnification image of (**b**). The scale bar is 600 μm. (**d**) Low-magnification SEM image of the 2-layered fishnet MoSM array. The scale bar is 600 μm. (**e**) Magnified SEM image of the red dotted box in (**d**). The white arrow indicates the slightly misaligned stacking of the MoSMs. The scale bar is 140 μm.

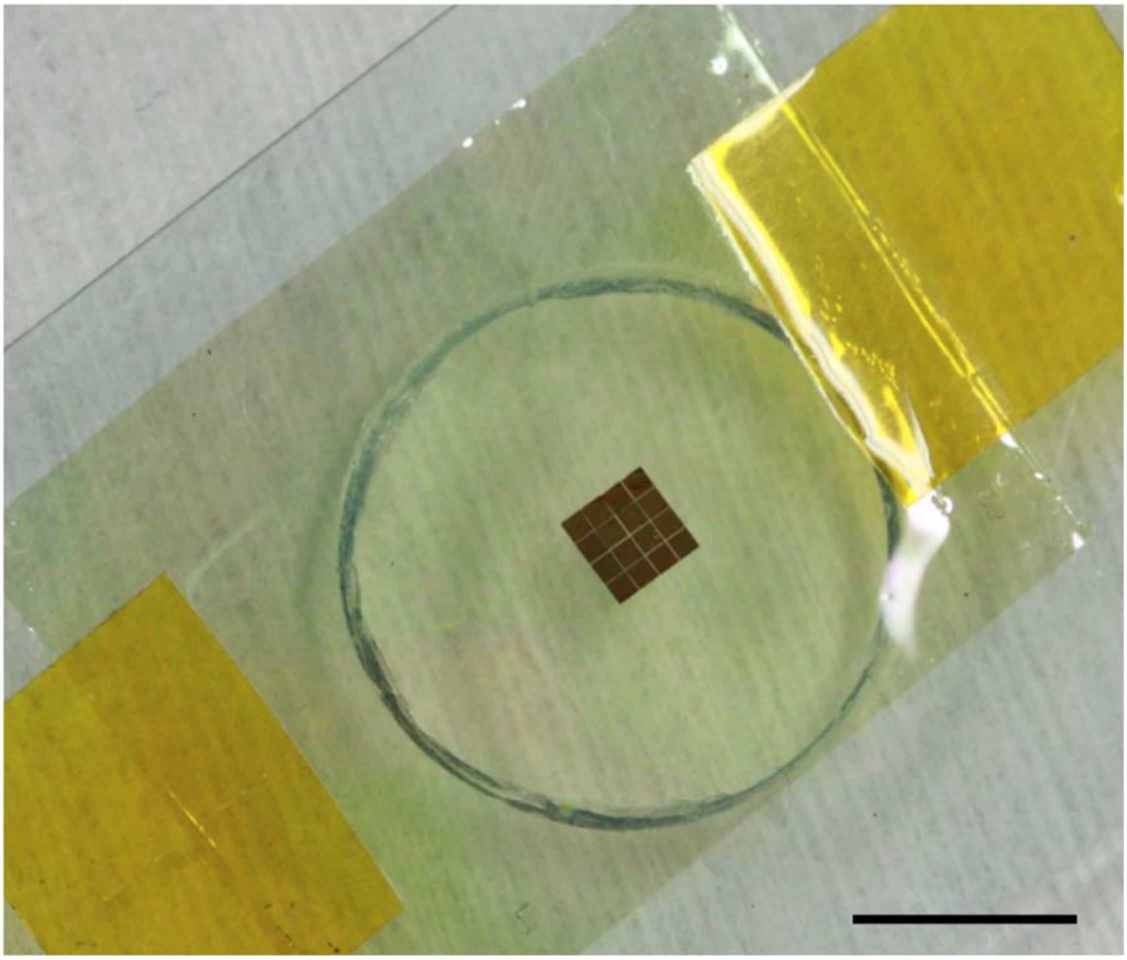

Supplementary Figure 16: **PI-encapsulated 3D metamaterials that were developed using modular transfer printing.** The scale bar is 5.0 mm.

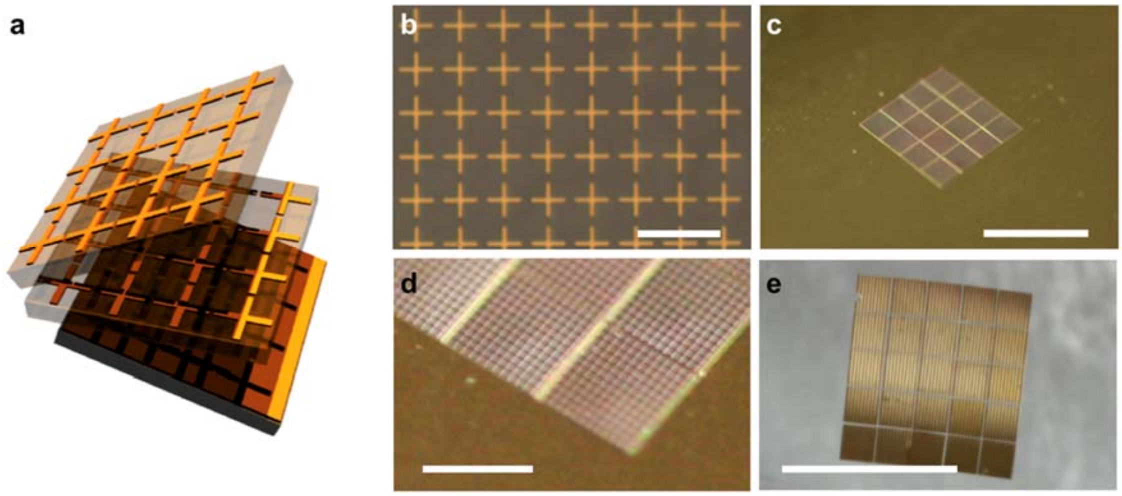

Supplementary Figure 17: **Attachment of metamaterial absorbers onto various substrates via 3D modular transfer printing** (**a**) Schematic of 3D modular transfer printing of each individual building block that was required for the construction of the metamaterial absorber (by stacking MoSMs with metallic cross patterns and a flat Au/Si membrane), (**b**) Top-view OM image of the MoSM with metallic cross patterns. The scale bar is 100 μm. (**c**) Image of a 4 x 4 MoSM array that was printed onto a flat Au film. The scale bar is 2.5 mm. (**d**) Macroscopic image obtained by magnifying the image shown in (**c**). The scale bar is 600 μm. (**e**) Macroscopic image of the metamaterial absorber that was assembled onto a THz-transparent PDMS substrate. After 3D modular transfer printing of the metamaterial absorber, the initially THz-transparent PDMS changed into a THz-absorbing material. The scale bar is 3.1 mm.

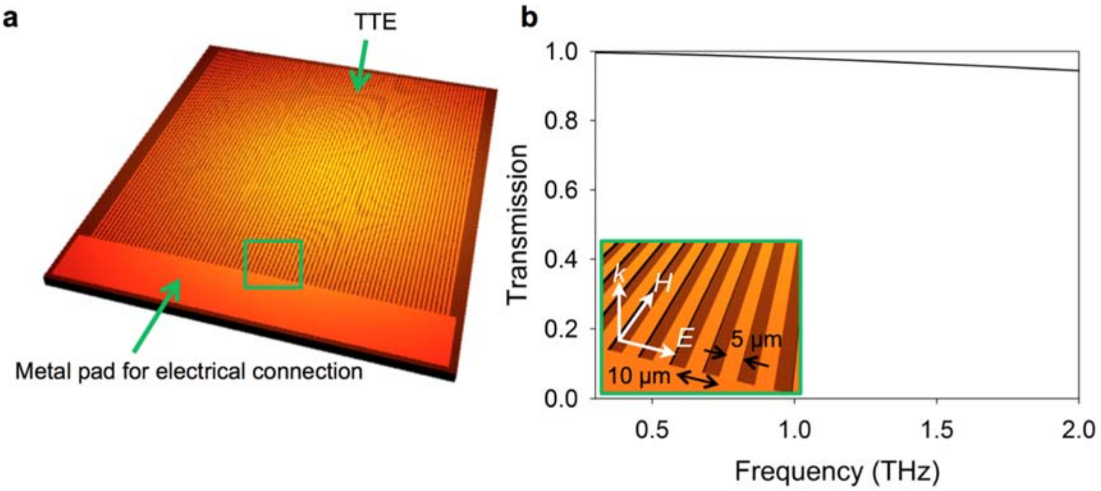

Supplementary Figure 18: **THz transparent electrode (TTE)** (**a**) Schematic rendering of the TTE. The designed TTE for this work consisted of a metallic (Au) microwire array with a width of 5 μm width and a periodicity of 10 μm. For the modular assembly, the TTE was developed on a PI layer via photolithography and metal lift-off. (**b**) The simulated THz amplitude transmittance through the TTE (inset shows the structural dimension). For this THz transparency, the *E*-field of an incident THz wave should be aligned with the grating vector of the microwire array.

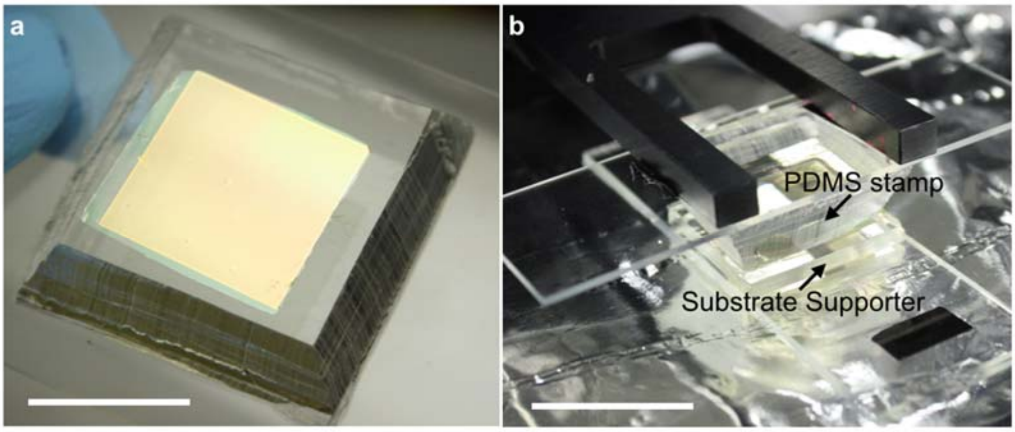

Supplementary Figure 19: **Printing of TTE for the assembly of electrically-gated graphene metadevices** (**a**) Image of a PDMS stamp used for the printing of TTE. The scale bar is 1.5 cm. (**b**) Captured image of the printing process of TTE in the fabrication of electrically-gated graphene metadevices. The scale bar is 5.0 cm.

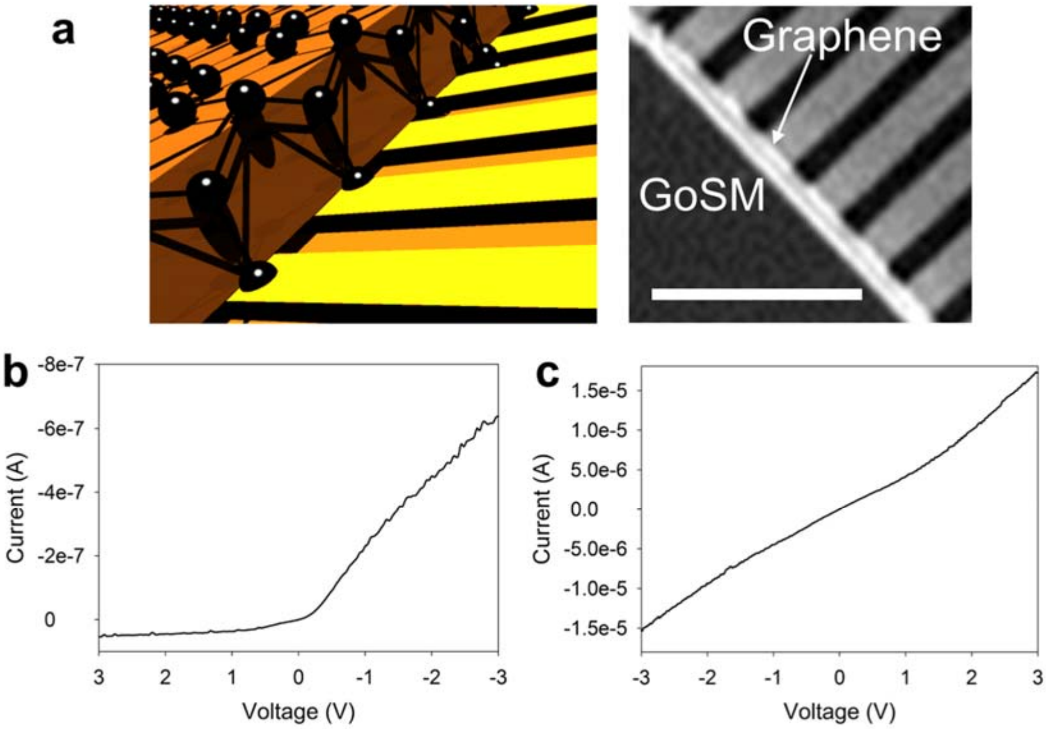

Supplementary Figure 20: **Ensuring electrical contact between the graphene/Si membrane and TTE** (**a**) Schematic and SEM image of GoSM that was printed onto TTE. Graphene wrapped around the sidewall of the Si membrane during the fabrication of GoSM (see Supplementary Fig. 3); thus, graphene was in direct contact with TTE. The scale bar is 30 μm. (**b**) I-V curve between the two terminals of TTE on which six bare Si membranes (without graphene) were printed (i.e., Schottky contact), (**c**) I-V curve between the two terminals of TTE on which six GoSMs were printed (i.e., Ohmic contact). These results indicate that graphene on a Si membrane can electrically contact TTE via 3D modular transfer printing.
